# Who Is *Dermanyssus gallinae*? Genetic Structure of Populations and Critical Synthesis of the Current Knowledge

**DOI:** 10.3389/fvets.2021.650546

**Published:** 2021-05-28

**Authors:** Lise Roy, Annunziata Giangaspero, Nathalie Sleeckx, Øivind Øines

**Affiliations:** ^1^CEFE, University of Montpellier, CNRS, EPHE, IRD, Univ Paul Valéry Montpellier 3, Montpellier, France; ^2^Department of Agriculture, Food, Natural Resources, and Engineering (DAFNE), University of Foggia, Foggia, Italy; ^3^Experimental Poultry Centre, Geel, Belgium; ^4^Norwegian Veterinary Institute, Ås, Norway

**Keywords:** *Dermanyssus gallinae*, haplogroups, mitochondrial DNA, CO1, tropomyosin, NUMTs, one-health, epidemiology

## Abstract

Despite the economic and animal welfare importance of the Poultry Red Mite *Dermanyssus gallinae*, its genetic structure has been studied in a scattered way so far. The prophylaxis and control of such a globally distributed ectoparasite can be significantly improved by understanding its genetic population structure (composition in species and intraspecific variants). The present study aims to establish a rigorous framework for characterizing the neutral genetic structure of *D. gallinae* based on a literature review combined with an integrative analysis of the data available in GenBank on population-level nucleotide sequence diversity supplemented by a new dataset. The integrative analysis was conducted on sequence data extracted from GenBank coupled with new sequences of two fragments of the mitochondrial gene encoding Cytochrome Oxidase I (CO1) as well as of an intron of the nuclear gene encoding Tropomyosin (Tpm) from several PRM populations sampled from European poultry farms. Emphasis was placed on using the mitochondrial gene encoding CO1 on which the main universal region of DNA barcoding in animals is located. The species *D. gallinae sensu lato* is a species complex, encompassing at least two cryptic species, i.e., not distinguishable by morphological characters: *D. gallinae sensu stricto* and *D. gallinae* L1. Only *D. gallinae s.s*. has been recorded among the populations sampled in poultry farms worldwide. Current knowledge suggests they are structured in three mitochondrial groups (haplogroups A, B, and C). Haplogroup A is cosmopolitan, and the other two present slightly contrasted distributions (B rather in the northern part of Europe, C most frequently found in the southern part). Recent data indicate that a dynamic geographic expansion of haplogroup C is underway in Europe. Our results also show that NUMT (nuclear mitochondrial DNA) pseudogenes have generated artifactual groups (haplogroups E and F). It is important to exclude these artifact groups from future analyses to avoid confusion. We provide an operational framework that will promote consistency in the analysis of subsequent results using the CO1 fragment and recommendations for future analyses.

## Introduction

A good understanding of the genetic structure of the populations of any parasite is a crucial prerequisite to optimize its prophylaxis and control. Indeed, the development of management tools can be improved if we know whether one or several species are involved in the infestations and if we trace the routes of dissemination of the populations. The tremendous economic, health, and animal welfare importance of the Poultry Red Mite (PRM), *Dermanyssus gallinae* (DeGeer, 1778), coupled with recurrent prophylactic and control failures, highlight the need for knowledge on the genetic structure, and population dynamics of this parasite. However, substantial gaps have been identified by the collaborative work of the COST COREMI FA1404 Action (https://www.cost.eu/actions/FA1404/#tabs|Name:overview) and Discontools.eu (https://www.discontools.eu/database/112-poultry-red-mite.html). Consequently, efforts in improving these areas are needed. Indeed, not only is PRM one of the under-studied parasites of economic importance, but the few published molecular studies of PRM come from the work of research teams belonging to different disciplinary fields (veterinary and medical parasitology and microbiology, pharmacology, evolutionary biology, taxonomy etc.,). They are published in journals from various disciplinary fields, which makes the pooling of results delicate. Four studies published between 2009 and 2011 (1–4) had established the first basis for the characterization of genetic groups within the genus *Dermanyssus* Dugès, 1834, with a focus on *D. gallinae*. Only one was published in a veterinary journal (3). Recently, several studies on the genetic structure of *D. gallinae* in layer farms in different regions of the world, mainly from Europe, have been published. Based on fragments of the gene encoding Cytochrome Oxidase I (CO1), some of them revealed genetic groups whose biological status (species or intraspecific variants) had not been determined. These groups could either be biological species in the sense of Mayr (5), i.e., groups of individuals that may interbreed with each other but not with different populations (groups i), or be intraspecific variants, interfecund with other populations of *D. gallinae s.s*. (groups ii), or constitute illusory groups, resulting from the presence of pseudogenes in the dataset (DNA sequences treated as homologs of others when they are not) (groups iii). The need to establish a standardized framework allowing an optimized and harmonized approach to analyze future molecular data on PRM guides the present study. This will improve our understanding of the problems associated with PRM infestations as it will allow more data to be compared across specific studies.

Let's start by laying the theoretical background for the exploration we propose to carry out. The formation of biological species in the sense of Mayr (5) (groups i) and of intraspecific variants (groups ii) result from the same evolutionary process, speciation, at more or less advanced stages [see (6) and lexical notes in (4)]. The genotypic composition of populations is constantly changing over time due to random events (mutations, genetic drift), and natural selection. The interruption of gene flow by any barrier that prevents cross-breeding between populations (e.g., geographical isolation) inexorably leads from generation to generation to their genotypic differentiation and consequently to phenotypic differences affecting various characteristics (morphology, physiology, behavior, etc.). After a certain time (numerous generations), reproductive incompatibility (a complete lack of reproduction between the individuals or reproduction resulting in infertile progeny) may appear and the entities thus differentiated are no longer able to exchange genes even if the barrier can be broken (i.e., through new contamination routes or movements by hosts): these end up as separate Mayr's species (5). But before complete separation occurs, such a break in barriers can lead to highly differentiated populations exchanging genes again [speciation reversal between incipient species (7)]. Deciding between the two above categories is far from evident since determining whether reproductive incompatibility has occurred between populations is practically impossible. We typically look for clues of disruption within a continuum of variation by bringing together different lines of evidence [molecular phylogenies, morphological analyses, ecological, and behavioral observations, etc., (8)]. Identifying these clues is a significant issue in pest management as considerable differences in pathogenicity, pesticide resistance, vectorial capacity, etc., can be associated with different pest species (or incipient species) as a result of the absence of gene flow between each another for many generations, regardless of whether or not they exhibit detectable morphological differences.

Cryptic species are populations that are reproductively isolated from others (= Mayr's species) but for which no morphologically discriminating characters have been identified (9). Nevertheless, they are likely to differ in various non-morphological characteristics as much as from closely related species that may be morphologically distinguished (9) and that may be present in the same environment. For example, aspergillosis agents were considered multi-resistant to antifungal agents before it was discovered that they were composed of different cryptic species, each with a narrow resistance spectrum (10). The distinction between these morphologically identical variants is clinically needed to treat the induced pathology (10) properly. Cryptic speciation has been widely described, especially in taxa of tiny invertebrates such as mites after molecular tools have been employed, leading to an understanding of a grossly underestimated diversity (11).

In addition, pseudogenes may confuse the information provided by a single region (groups iii): particularly in mitochondrial analyses, where these non-functional variants of the region of interest can be amplified with mitochondrial DNA and aligned as if they were orthologous sequences (12–14). These paralogous sequences result from past incorporation of mitochondrial DNA copies into the nuclear genome and are known as NUMTs for “nuclear mitochondrial DNA”. They do not reflect the same evolutionary history as the targeted genomic region and can generate artifact genetic groups in analyses (with percentages of divergences from conspecifics typically greater than intraspecific values). This happens because NUMTs lose their functionality when integrated into the nuclear genome and are therefore freed from selective pressures usually experienced by functioning genes. In addition, they are subjected to different molecular repair mechanisms after being incorporated into the host nuclear DNA. Distinguishing cryptic species (groups i) within mitochondrial variants from the artifact groups produced by NUMTs (groups iii) is, therefore, crucial to clarify the genetic structure of a parasite and to advance the understanding of its epidemiology.

**Figure 1:**
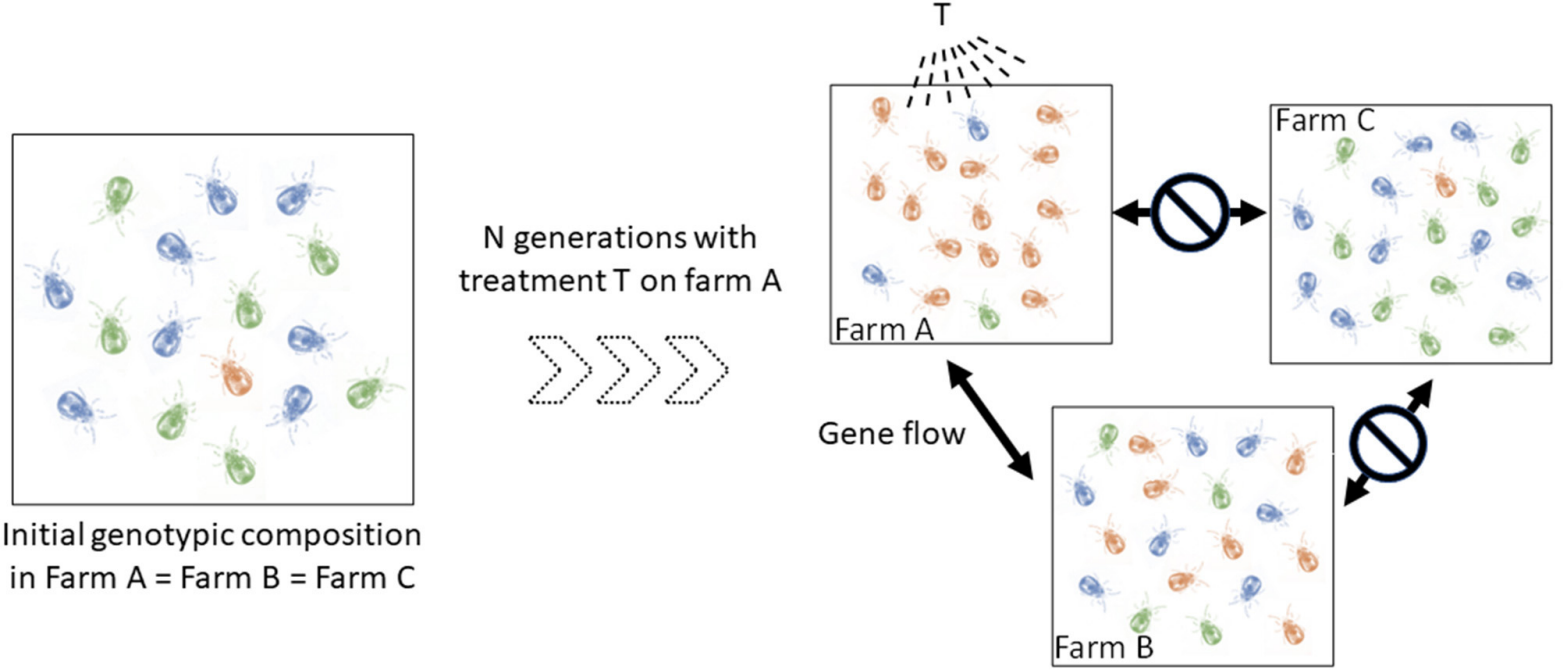
Schematic illustration of the implication of spatio-temporal dynamics in the diffusion of a dominant resistance to antiparasitic agents. Let consider three hypothetical farms, two of which are interconnected (farm A, farm B), i.e., they exchange mites regularly, and one isolated farm (farm C). Left, the blue and green genotypes are sensitive to the T treatment (only sensitive alleles), the orange is resistant to it (i.e. any genotype containing the resistant allele) (to simplify, we start from identical initial genotypic compositions in the three farms, with the resistant genotypes (i.e. any genotype containing the dominant allele) being very infrequent). *Right*, genotypic composition after a certain number of generations of mites having undergone the selective pressure of the T treatment in farm A and having received no treatment in farms B and C. In farm A, mites carrying the resistant allele (orange) were favored by the T treatment and thus transmitted this allele to a large number of offspring. The resistant genotypes thus became the majority. Because of the gene flow between farms A and B and the isolation of farm C, this resistant allele has increased in frequency in farm B and not in farm C. If farmers B and C start applying the T treatment at this time, the former is likely to experience treatment failures very quickly, while the latter will be able to treat effectively for some time.

In addition, a better understanding of the spread routes between populations of mites (= infestation routes between farms) is also central to progress on the knowledge of their epidemiology. Individuals from two connected populations have almost as much chance of reproducing with each other as within one of them. In contrast, individuals in less connected populations have more chance to reproduce with individuals inside their own population. Such a simple reduction of gene flow can produce population differentiation (groups ii) whose effect in terms of clinical management is not negligible. Identifying the direction and location of gene flow amongst infested farms can shed important light on infection events and the origin of infections. Importantly, this information is relevant for making prophylactic recommendations. This also has direct relevance for predicting the evolution and spread of anti-parasitic resistance, should that appear in the mite population. The degree of connection between farms is expected to vary according to a number of parameters closely related to poultry transfer routes, including logistic networks, geographical proximity, and sanitation regimes. To illustrate, if the population in farm A has developed resistance to T treatment and is strongly connected to farm B's and not connected to farm C's, genotypes with resistance to T treatment are more likely to spread to the farm B than to farm C, especially if the resistant allele is dominant (Figure 1). Therefore, if farmers B and C start applying the T treatment for the first time on their farm at the same time, farm B is likely to experience treatment failures very soon, while farm C will take much longer to experience this problem. Of course, the kinetics of resistance emergence in farms B and C will also be modulated by the fitness cost of the resistance to T treatment, but the dynamics of the development of resistance to the treatment may be strongly different between these farms. Mapping in time and space the intraspecific variants (possible groups ii) allows the inference of the spread routes of the organisms [e.g., (15, 16)].

Now let us come to the state of the art concerning Poultry Red Mites. To understand epidemiological patterns of *D. gallinae* infestations and its resistance to parasiticides, a correct knowledge of any boundaries between species (groups i: who is who?) is necessary, as well as of epidemiological dynamics (groups ii: who goes where?), which requires the selection of relevant genetic markers (prevent artifacts due to groups iii). Regarding the species composition of the genus *Dermanyssus*, Moss (17, 18) provided during the 20th century the only in-depth taxonomic study of the entire genus *Dermanyssus* using morphology alone, with helpful dichotomous keys to the 18 species described so far. The genus *Dermanyssus* now contains 25 described species and is divided into two subgenera, *Dermanyssus* and *Microdermanyssus* (1). The subgenus *Dermanyssus* is divided into two groups of species: the *hirsutus* group and the *gallinae* group (17). Here we focus on the *gallinae*-group, which contains the economically important *D. gallinae* along with 14 other species. Moss (18) claimed that diagnostic morphological characters were difficult to define and explicitly urged those seeking to identify mites of the genus *Dermanyssus* based on morphology to be cautious in the use of his key because of the extensive intraspecific and intrapopulation variation in the diagnostic characters that he retained (although after more than 10 years of careful examination). In their studies, Roy et al. (1, 2, 4) have focused on the interspecific delineation of the *gallinae*-group within the *Dermanyssus* subgenus by combining morphological and molecular analyses. The latter two studies (2, 4) questioned the fine lines by considering intra-population variations. Roy et al. (1) confirmed the difficulties of morphological identification, set the interspecific boundaries between widely divergent entities and solved some morphological issues by pointing out segregating (allowing a species diagnosis) and overlapping (blurring the distinction between species) characters within genetic groups defined by phylogenetic analysis of mitochondrial and nuclear sequence alignments. Roy et al. (1, 19) reported several mitochondrial groups as possible species presenting a less deep divergence but did not validate them due to lack of corroboration by more than one line of evidence recommended by DeSalle et al. (8). Roy et al. (2) and Roy and Buronfosse (4) have partly filled this gap and confirmed some interspecific boundaries (cryptic species *D. gallinae* L1) but not all (clade E, isolate JOW). Interestingly, the haplotypic and nucleotidic diversities of both mitochondrial and nuclear regions were found to be substantially higher within *D. gallinae* populations compared to other species of the genus *Dermanyssus*, suggesting recurrent events of hybridization between incipient species in the pest species (1, 4). Regarding the population composition within *D. gallinae*, seven articles deal with the population structure within *D. gallinae* based on mitochondrial DNA sequences with the aim to answer questions related to the management of the parasite in the poultry industry (3, 4, 20–24) whereas, three studies used mitochondrial DNA sequencing to distinguish two strongly differentiated genetic pools within *D. gallinae* in a human pathology context (25–27).

The objective of the present study was to summarize and critically review the knowledge on the mite population and species structure obtained so far using Sanger-sequencing technology in the Moss' *gallinae*-group within the genus *Dermanyssus* with a focus on the species of economic and animal welfare importance, namely *D. gallinae*. This work is based both on a review of the literature and on an integrative analysis of the DNA sequence data associated with the publications, supplemented by the acquisition of new data. In particular, we will take the opportunity to clarify the groupings delineated so far within *D. gallinae* based on CO1 gene by establishing synonymies between them and by spotting the artifact groups generated by pseudogenes. Such critical elucidation - with a didactic approach -, supplemented by the analysis of new molecular data and by recommendations, will help researchers to provide a more efficient standardized handling of molecular tools, able to better answer questions related to the biology and epidemiology of *D. gallinae* and similar organisms.

## Materials and Methods

### Literature Review

The available literature on the taxonomy of the genus *Dermanyssus* and the neutral genetic structure of *D. gallinae* was reviewed by searching research papers through three electronic databases (Scopus, Web of Science and Google Scholar) until December 2020 with no time limits. A review has been realized by highlighting the historical development of the successive discoveries and the unsolved questions.

### Sequence Data

**Figure 2:**
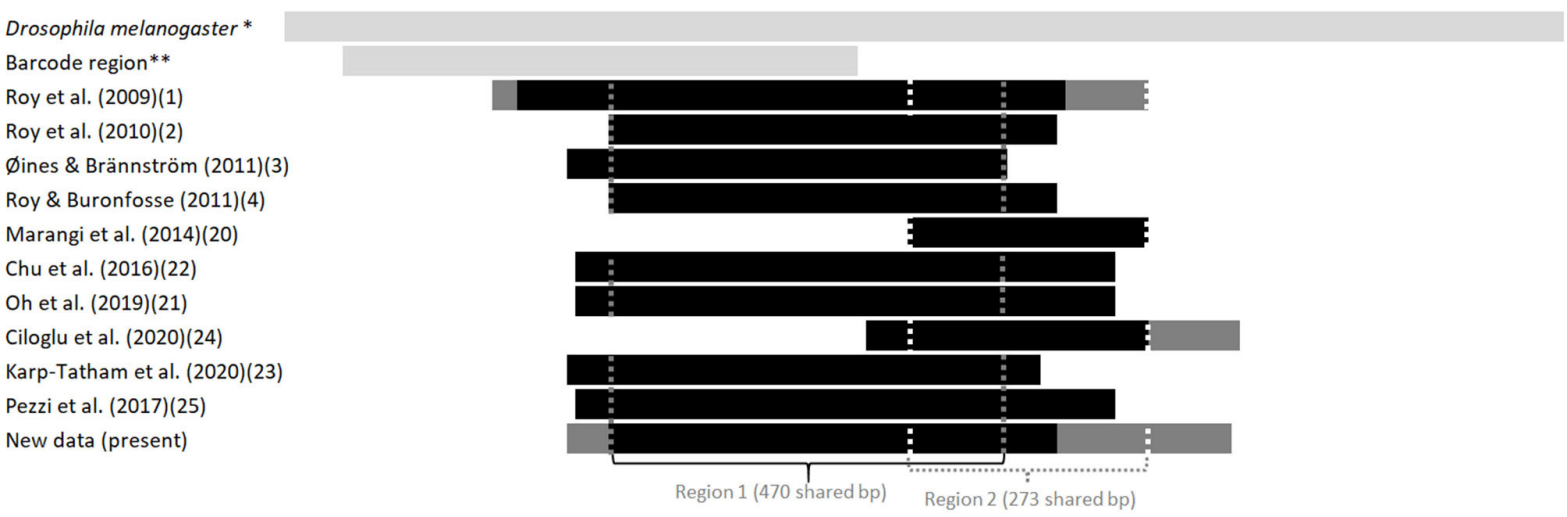
Length and position in relation to the *Drosophila*'s full sequence of fragments of the gene coding for CO1 available in the GenBank from various studies on the genus *Dermanyssus*. The first light gray bar at the top illustrates the sequence length of the complete CDS gene in *Drosophila melanogaster*, taken as a reference, and the second one shows the length and relative position of the barcoding region. The following bars represent the positions of the sequences studied in relation to this reference sequence. Light gray, reference sequences (from studies not considered here), black, all sequences in a study; dark gray, longest sequences among sequences of various sizes (when a longer fragment has been sequenced in a part of the mites in a study). The vertical dotted lines delimit the sequences used in the two CO1 alignments analyzed in the present study (namely “region 1” and “region 2”). *NCBI Reference Sequence: NC_024511.2. ** Based on the GenBank sequence #AY956082.

The mite sequences included in the studies listed in Table 1 were collected from the GenBank database (per October 2020) and an unpublished repository (unpublished data, LR). The new molecular data were acquired from mites collected from bird farms (Table 2). The mites were individually processed. The DNA extraction from each individual was performed with the REDExtract-N-Amp Kit (Sigma-Aldrich, Saint Louis, MO, USA), following the manufacturer's recommendations after a rough crushing with a pipette cone. One or two of the regions of the CO1 gene defined in Figure 2 were amplified in all individuals with the primer pairs shown in Table 3. To verify that the different primer pairs used in the various studies did not amplify different CO1 sequences in the same individual (NUMTs or heteroplasmia), some individuals were treated with two different primer pairs. To refine the understanding of the genetic structure at the borderline between sister species and intraspecific variants, we also amplified the intron n of the Tropomyosin (Tpm) coding gene in a subsample of these new mites (see Table 2) with the primers listed in Table 3. PCR was performed with a 11-μL total reaction mix and 2 μL of diluted DNA (up to 20 ng) in a Perkin-Elmer PE 9700 thermal cycler (Perking-Elmer, Waltham, USA). The reagent concentrations were 1 x PCR buffer (Qiagen), 0.04 U/μL Qiagen *Taq* Polymerase, 200 μM dNTPs, and 0.3 μM of each primer, and 3.4 mM of MgCl_2_ for CO1 and 1.5 for Tpm. DNA amplification was carried out as follows: initial denaturation at 94°C for 5 min, followed by 5 cycles of denaturation at 94°C for 30 s, annealing for 30 s at 48°C (CO1) or 56°C (Tpm), elongation for 1 min at 62°C (CO1) or 72°C (Tpm), 35 cycles of denaturation at 94°C for 30 s, annealing for 30 s at 52°C (CO1) or 56°C (Tpm), elongation for 1 min at 62°C (CO1) or 72°C (Tpm), and final extension at 62°C (CO1) or 72°C (Tpm), for 10 min. Negative controls (without DNA matrix) were included in all PCR runs and amplification products were verified by 1% agarose gel electrophoresis and Ethidium Bromide staining. Both strands of final PCR products were Sanger sequenced using a commercial sequencing-service (MWG Eurofins Operon sequencing laboratory, Germany). The chromatograms' quality was checked using Chromas v2.6.6 (http://technelysium.com.au/wp/chromas/) and were manually corrected in case of misinterpreted peaks (N on a clear peak or bridges produced by local dye saturations), and the primer region was omitted. The sense and antisense sequences obtained from the PCR-products were assembled using CodonCode Aligner (CodonCode Corporation, www.codoncode.com). For Tpm, a special treatment was performed using R (29) to distinguish alleles in heterozygotes without going through a resequencing with internal primers as carried out in (4): analysis of .ab1 files with the sangerseqR (30) and Biostrings (31) packages to transform the double peak sequences into consensus sequences with IUPAC codes, then computer separation of the two alleles using Indelligent v.1.2 (32). This allowed obtaining rather coarse data which were clarified by comparing the alleles obtained in homozygotes with the alleles distinguished by Indelligent and going back and forth between alignment and chromatograms.

**Table 1:** Studies selected for integrative analysis.

**Reference**	**Co1 primer pairs for *D. gallinae* s.l**.	**Geographic range**	**Sampling date**	**Taxonomic range**
Roy et al. (1)	COF1 + RQCOIR, CO1F4 + RQCOIR, COF1bis + RQCOIR, COF1bis + ObCOIFrev, CO1LCF + RQCOIR	France/Europe/USA	2006–2008	*Dermanyssus*
Roy et al. (28)	Same pairs without COF1 + RQCOIR and with SKPO + RQCOIR in addition	France/Europe/USA	2006–2008	*Dermanyssus*
Roy et al. (2)	Same pairs as Roy et al. (28)	France/Europe	2006–2009	*Dermanyssus*
Øines & Brännström (3)	FCOIDG + RCOIDG	Norway/Sweden/Europe	2004–2009	*D. gallinae s.s*.
Roy & Buronfosse (4)	Same pairs as Roy et al. (28)	France/Europe/Australia/Brazil	2007–2010	*D. gallinae s.l*.
Marangi et al. (20)	COX1F + COX1R	Italy/Europe	2011	*D. gallinae s.s*.
Chu et al. (22)	CO1Fyuw114 + CO1Ryuw114	Japan	2005–2012	*D. gallinae s.s*.
Pezzi et al. (25)	CO1LCF + RQCOIR	Italy	2015	*D. gallinae s.l*.
Oh et al. (21)	CO1Fyuw114 + CO1Ryuw114	Korea	2018–2019	*D. gallinae s.s*.
Ciloglu et al. (24)	COX1F + COX1R	Turkey/Europe	2016–2017	*D. gallinae s.s*.
Karp-Tatham et al. (23)	CO1Fyuw114 + CO1Ryuw114	UK/Japan/Europe	2017–2018	*D. gallinae s.s*.
COF1 (region 1, forward: 5′-ATCGGAGGATTCGGAAACTG-3′)		COX1R (region 2, reverse: 5′-TACAGCTCCTATAGATAAAAC-3′)	
CO1F4 (region 1, forward: 5′-CACCTGACATGGCTTTCCCACGAT-3′)		CO1Fyuw114 (region 1, forward: 5′-AGATCTTTAATTGAAGGGGG-3′)	
COF1bis (region 1, forward: 5′-CTGCACCTGACATGGCTTTCCCAC-3′)		CO1Ryuw114 (region 1, reverse: 5′-AAGATCAAAGAATCGGTGG-3′)	
CO1LCF (region 1, forward: 5′-GAAAGAGGAGCAGGCACTGG-3′)		RQCOIR (region 1, reverse: 5′-CCAGTAATACCTCCAATTGTAAAT-3′)	
SKPO (region 1, forward: 5′-CTTTTTAGATCTTTAATTGAAA-3′)		ObCOIFrev (region 1, reverse: 5′-GTGGGAATHGCAATAAT-3′)	
FCOIDG (region 1, forward: 5′-CATTAATATTAACTGCACCTGACA-3′)		RCOIDG (region 1, reverse: 5′-CCCGTGGAGTGTTGAAATTCATGA-3′)	
COX1F (region 2, forward: 5′-TGATTTTTTGGTCACCCAGAAG-3′)		COIGOR (region 1, reverse: 5′-GTTGGAATTGCAATAAT-3′)	

*We reviewed all available studies that have investigated the genetic structure of D. gallinae populations using CO1 sequences. All of these studies extracted DNA from the mites individually and amplified at least one region of CO1. However, data entry into the GenBank was not performed in exactly the same way. Most authors associated each sequenced mite with a unique accession number. However, Ciloglu et al. (24) submitted haplotypes individually (only one accession number per haplotype), Marangi et al. (20) submitted part of the obtained sequences without specifying the selection criteria and Oh et al. (21) sequenced DNA from pools of mites instead of mite individuals, and they submitted one accession number per pool. The nucleotide sequences of each primer are shown at the bottom of the table*.

**Table 2:** Information on new data.

**Sample ID**	**Sampling range**	**Co1**		**Tpm**	**Origin**
		**Region 1**	**Region 2**		
DIN	p	a	d	e	France (layer farm)
JOUV	p	a	d	e	France (layer farm)
GZ	p	a	d		France (backyard henhouse)
BE01	p	a			Belgium (layer farm)
BE02	p	a			Belgium (layer farm)
FR01	p	a			France (layer farm)
FR02	p	a			France (layer farm)
FRNP01	p	a			France (layer farm)
H	p	b′,b^′′^,c		e	France (layer farm)
SPT2018	p	a			France (layer farm)
SPT2020	p	a			France (layer farm)
CRZA	(p)	(a)	d		France (layer farm)
Quail	i	a			Belgium (lab quails)
Felska	i		d		Poland (layer farm)
MONT	i	b'			France (flat in a city)

*The mites were collected directly from commercial layer farms, except for GZ (private backyard poultry) and Quail (laboratory quail farm). The sampling size is considered to be at population level (p) when >15 individuals were collected from different locations in the henhouse. Otherwise, it is considered to only provide individual-level information (i). In the columns CO1 and Tpm, a letter indicates that there has been sequencing on the considered fragment and refers to the primer pair used (see Table 3)*.

**Table 3:** Primer pairs used to obtain new sequences in the present study.

**Genomic region**	**Primer pair**	**Forward primer**	**Reverse primer**
Co1 (region 1)	a	CO1LCF	RQCOIR
	b	CO1LCF	COIGOR
	b′	SKPO	COIGOR
	b^′′^	SKPO	RQCOIR
	c	RhipiCOIF	TyphloCOIR
Co1 (region 2)	d	COX1F	COX1R
Tpm (intron n)	e	TropoF5bisF	TropoF5bisR

*Except for primer pairs c and e, the nucleotide sequences of each primer are shown below Table 1. Here are the nucleotide sequences of primer pairs c and e: RhipiCOIF, 5'-CGAATAAATAATATAAGATTTTGA-3'; TyphloCOIR, 5'-GCTAATCAAGAAAAAATTTTAAT-3'; TropoF5bisF, 5'-TCGAGCACAGGAACATCACTG-3'; TropoF5bisR, 5'-AGTCTCGGCACGGTCTTCA-3'*.

### Processing of DNA Sequences

The sequences obtained from the databases, or from the newly generated mite collections were aligned in Seaview v5.0.4 (33) with the Clustal omega program for CO1 and Muscle program for Tpm. Each alignment was checked and corrected by hand in case of obvious local misalignment.

Given the large amount of available CO1 sequence data obtained with different sampling designs and based on primers positioned at different locations on the gene, we chose to work on haplotypes defined in two regions separately to conduct our integrative analysis. A haplotype is a unique sequence of nucleotides on a specific gene region. For mitochondrial genes, each individual carries one haplotype (two for nuclear genes) and shares it or not with other individuals. Isolating them from more or less numerous copies within populations is an essential preliminary step in population genetics to infer the evolutionary history of the gene portion concerned and compare the frequency of haplotypes between populations. By definition, the different haplotypes can only be distinguished from each other over a gene region for which data are available for all aligned sequences. When aligning together incomplete sequences (longer or shorter portions) of a given gene fragment, the non-overlapping flanking regions prevent exhaustive pairwise comparisons between all the sequences. Removing the incomplete flanking parts of the alignment makes it possible to obtain haplotypes for all the sequences. However, fewer varied nucleotide sites are likely to be encountered on a short fragment than on a longer one, resulting in a reduced proportion of different haplotypes detected in the population. Therefore, this approach is likely to provide little information on available diversity when the overlapping region is short. Alternatively, removing incomplete sequences provides a more resolved picture of the genetic diversity by allowing a more significant proportion of pairwise differences to be detected, but this reduces the benefit of large datasets. Here, to integrate all available data into the analysis while maintaining a sufficient haplotype length, in view of the location of the overlapping areas in the collected data set (Figure 2), we have chosen to distinguish two regions (1 and 2) and treat them independently. For the individuals with the two CO1 “regions” sequenced (isolates called DIN, JOUV, and GZ; Table 2), the overlapping part was carefully examined (chromatograms + alignments) to verify *in silico* that the pairs of primers amplified the same haplotype for each individual and not different copies.

The three different datasets (two CO1 regions and one Tpm region) were isolated in Seaview in fasta format. The nucleotide sequences corresponding to the CO1 sequences obtained were translated into amino acid sequences and displayed in Seaview. Haplotypes were individualized with DnaSP v6.12.03 (34) after conversion in Seaview. The haplotype file was then converted in DnaSP to allow phylogenetic analysis in MEGA X v10.1.8 (35). Prior to phylogenetic analysis, each alignment has been the subject of a search for the best nucleotide substitution model to be used in the phylogenetic calculations using MEGA X. The phylogenetic analysis of the alignments obtained was conducted using the maximum likelihood method in MEGA X for each dataset with the model that had received the lowest AICc value (AIC with a correction for small sample sizes) in the previous search.

In the MEGA X tree editor, labeling directly on the selected CO1 tree (one for each “region”) of each haplotype from the information available in each study under scrutiny (assignment to a given group: clade, cluster and/or haplogroup) made it possible to evaluate the concordance between the different denominations and to establish the synonymies between the different denominations encountered in the literature to designate genetic groups within *D. gallinae*. On this basis, the different haplotypes identified in the present integrative analysis were assigned to clearly delineated mitochondrial groups within *D. gallinae*. The CO1 haplotype alignment nexus files of *D. gallinae s.l*. were then supplemented with geographic information (coded as traits). They were subjected to a haplotype network reconstruction using the minimum spanning network (MSN) method (36) in PopART software (37).

## Results

### Summary of Data From the Literature

#### Molecular Markers

To date, investigations on the genetic structure of the genus *Dermanyssus* and/or the species *D. gallinae* have been carried out based on five molecular markers obtained by Sanger sequencing: two mitochondrial genomic regions, i.e., fragments of 16S rRNA (1, 2, 22, 27), fragments of the gene encoding CO1 (1–4, 19–26, 28) and three nuclear genomic regions, i.e., the internal transcribed spacers (ITS), including fragments of ITS1 and ITS2 and nuclear ribosomal subunits 18S, 28S rRNAs, and 5. 8S (1–3, 20–22), elongation factor 1-α (EF-1) (2) and an intron of Tropomyosin (Tpm) (2, 4).

The four most commonly used markers (mitochondrial 16S rRNA and CO1, nuclear ITS, and to a lesser extent, EF-1α) in taxonomic and phylogeographic studies provide very heterogeneous information within the genus *Dermanyssus* (1, 2), and different phylogenetic resolutions. Only CO1 was found to be truly informative in measuring intraspecific diversity in the genus *Dermanyssus* (% sequence pair differences of up to 9%) but was followed quite closely by a fragment of the gene encoding the mitochondrial ribosomal 16S rRNA. The latter shows sufficient variation to detect intraspecific structures or at the inter-/intraspecific interface (22, 27). Notably, the ITSs (ITS1 and ITS2) are very poorly informative due to the lack of intraspecific (and sometimes even interspecific) variation^1^. EF-1α, the second nuclear marker tested, was excluded from useful markers for exploring the genus *Dermanyssus* due to signs of paralogy (2).

The fifth marker used, an intron of the nuclear gene encoding Tropomyosin (Tpm), is an unconventional marker of rapid evolution, specifically developed by (2) to resolve taxonomic issues within the genus *Dermanyssus* despite the shortcomings of the two nuclear markers above. In search of a nuclear marker of rapid evolution, Roy et al. (2) amplified an intron of the gene encoding the Tpm protein whose coding regions (CDS) were available from the GenBank (accession number: AM167555.1; intron between nucleotides #495 and #496). The usefulness of this intron for exploration of genetic groups at the interface between inter- and intraspecific levels in *Dermanyssus* was found to be important as it shows a high variation of the same order of magnitude as CO1 in this species [36 CO1 haplotypes with up to 9% divergence, 39 Tpm alleles with up to 6% divergence in (2)]. In addition, more than three alleles were typically recorded within single populations (one farm or one bird nest), but only single or double sequences were encountered from single mite individuals, supporting the idea that the primers used amplify only a single orthologous locus (4).

Unlike highly variable mitochondrial genes (at least in the absence of NUMTs, as they are haploid; see above), one disadvantage associated with high variation of nuclear genes when using Sanger sequencing, is the presence of heterozygotes (individuals with two haplotypes; see the material and methods section), which results in double peak successions. To solve this problem, Roy et al. (2, 4) took advantage of the recurrent presence of heterozygous indels (indels of fixed 3-7-bp sequences) across the intron n to separate the two alleles of each heterozygous individual, as follows: (i) mapping of these indels based on sequence alignment of homozygous individuals allowed the definition of internal primers targeting the alleles with and without the different indels; (ii) the first heterozygous indel in each of the two directions was located on chromatograms from heterozygous individuals; (iii) two new sequencing reactions were conducted in each direction for each heterozygous individual to obtain the totality of the sequences of each allele. Although, somewhat tedious and expensive, this procedure has allowed refining the use of a nuclear marker to assess interspecific boundaries and to clarify the phylogenetic relationships within the genus *Dermanyssus* (19), but also to explore the genetic structure of *D. gallinae* populations (4) as well as, more recently, the genetic structure of mesostigmatic mite species belonging to the family Laelapidae (41).

#### Definition of Interspecific Boundaries in the Genus *Dermanyssus*

Roy et al. (1, 2) delineated the species of the genus *Dermanyssus* using a morpho-molecular total evidence approach, seeking concordant patterns between the distribution of morphological characters and both mitochondrial phylogenies and nuclear phylogenies. It is assumed here that species of the genus *Dermanyssus* all need males to develop generations (sexual reproduction at the population level), like the only species of the genus whose mode of reproduction has been studied (*D. gallinae*) as well as their close relatives *Ornithonyssus* spp. and *Ophionyssus natricis*. Indeed, the reproduction mode of all these mites is haplodiploid, i.e., males emerge from unfertilized oocytes and females from fertilized oocytes (42–44). To delineate sexually-reproducing species, the search for congruent clusters (clades) of individuals in nuclear and mitochondrial phylogenetic trees is crucial because, in most animals, the mitochondrial genome is transmitted by the mother (via the cytoplasm of the oocyte) whereas the nuclear genome is transmitted in a biparental manner (half by the mother and half by the father, with recombination). Consequently, within the species, because of the reticulated relationships between individuals, the evolutionary history (visualized as a phylogenetic tree) of a region of the mitochondrial genome (maternal lineage) is expected to be different from that of a region of the nuclear genome. On the other hand, between species, i.e., between entities that have not exchanged genes for a long time, the evolutionary history of the same two genomic regions is likely to be congruent. Consequently, while phylogenetic analysis of a single genomic region gives an idea of the genetic structure of a taxon, it does not allow us to identify unambiguously the points of divergence resulting from advanced speciation.

In a well-studied genus, i.e., where the interspecific boundaries have been rigorously defined on the basis of sufficient inter- and intraspecific sampling to provide a good representation of genetic variation, a gap between the largest intraspecific distance and the smallest interspecific distance is expected, referred to as the “Barcoding Gap” (45). Thus, the percentages of sequence divergence or genetic distances in pairs of individuals of the same species are generally lower than what is observed in pairs of individuals belonging to two different species. However, these values vary substantially between taxa, and there is no universal reference value for identifying interspecies boundaries based on mitochondrial topology alone, all the more when introgression occurs (46, 47). Therefore, to prevent confusion due to the presence of cryptic species and/or to NUMTs, searching in phylogenetic trees for the level of clustering of individuals that matches between mitochondrial and nuclear analysis is a reasonable way to establish interspecific boundaries [e.g., (48, 49)]. Indeed, it is expected that:

- the phylogenetic relationships between mitochondrial and nuclear sequences will be very different within the species;
- the node grouping all individuals of the species will be concordant (and not internal clades).

To observe this kind of pattern, it is necessary to work on mitochondrial and nuclear genomic regions that are sufficiently variable for the phylogenetic divergences to be informative in both phylogenetic analysis groups.

The morpho-molecular approach developed by (1) based on 46 morphological characters, two mitochondrial markers and one conventional nuclear marker (rRNA 16S, CO1, ITS) allowed a robust delimitation of the species described at that time and included in the *gallinae* group of the genus *Dermanyssus* (the other species groups were only studied morphologically) and the discovery of a species not then described: *Dermanyssus apodis* Roy, Dowling, Chauve & Buronfosse 2009. The boundaries between these species among the different populations studied are supported by the existence of segregating morphological characters (i.e., synapomorphies; stable within the species and presenting different states between species) and the concordance of groupings of individuals between mitochondrial and nuclear phylogenies. However, several strongly divergent mitochondrial lines, in the position of *D. gallinae*'s sibling group, were observed, the status of which could not be clarified due to a lack of variation in the nuclear marker ITS: clade E and clade F (= lineage L1; Table 4). Using the new Tpm marker (see above), interspecies boundaries among divergent mitochondrial groupings in morphologically indistinguishable *D. gallinae* mites could be explored (2). The F clade of (1), referred to as “special lineage L1,” was found to be a cryptic species. *Dermanyssus gallinae s.l*. is thus a complex of cryptic species, including at least *D. gallinae s.s*. and *D. gallinae* L1 (2, 4). In contrast, the mitochondrial haplogroup named “clade E” in (1) remained classified in *D. gallinae s.s*. because it shares its Tpm alleles with the other mitochondrial groups of *D. gallinae s.s*. (2).

**Table 4:** Correspondence of the different identifiers used in the studies included in this analysis to designate mitochondrial groups and their taxonomic positioning determined by comparing mitochondrial and nuclear data.

**Identifiers in the different studies**								**Taxonomic positioning**
**RO0**	**RO1**	**OB**	**RB**	**CHU**	**KT**	**CIL**	**Present**	
Clade E	Co21 & Co22						Co21-22	NUMTs
	JOW						JOW	NUMTs
	Lmt3	A	Lmt3	A	A	A	A	*D. gallinae s.s*.
	Lmt2	B	Lmt2	B	B	B	B	*D. gallinae s.s*.
	Lmt1	C	Lmt1	C	C	C	C	*D. gallinae s.s*.
clade F	L1	D	L1	D	D	D	D	*D. gallinae* L1
						E	E	NUMTs
						F	F	NUMTs
							H	NUMTs

*The identifiers placed on the same line refer to the same genetic groups. The study by (20) is not included because the phylogenetic reconstruction presented does not allow a correct assignment of sequences to the identified groups (see text). This lack of assignment was corrected in (24), who integrated part of the data from (20). The right-hand column (“present”) proposes to set the simplest identifiers for use. RO0, (2); RO1, (2, 19, 28); OB, (3); RB, (4); CHU, (22); KT, (23); CIL, (24). NUMTs, “nuclear mitochondrial DNA” (artifactual genetic group, not to be taken into account)*.

Finally, mites collected in farms show a particularly marked divergence between the Tpm alleles they carry (4). On farms, two to three nuclear Tpm alleles that are very distant from each other [first-level clusters in (4)] are frequently encountered with heterozygous status (often ‘Tro1' and/or ‘Tro2' and/or ‘Tro3' alleles). In wild avifauna, heterozygotes of *D. gallinae s.s*. generally carry much less distant alleles, assigned to a single of the three groups encountered in poultry farms (2, 4). This major divergence between alleles of *D. gallinae s.s*. in farms may be the result of multiple hybridization events between incipient species (secondary fertile contacts between populations that have been separated for a long time), perhaps due to the breaking down of geographical barriers by the long-distance transfers exerted by humans. Roy and Buronfosse (4) also reported some evidence of selection effects in the allelic composition of some farm populations as can be expected from pest control treatments and other hygiene activities (reduction in the number of Tpm alleles and CO1 haplotypes).

Three atypical mitochondrial groups (highly divergent from the others) have also been reported in mites morphologically conforming to *D. gallinae s.l*. These are the JOW haplogroup, found in a poultry farm in the USA (19) and the E and F haplogroups, found in poultry farms in Turkey and Italy (24). The lack of nuclear information for these mitochondrial haplogroups made it impossible to determine the level of reproductive isolation between these mites and the others (and thus to decide whether they are separate species, intraspecific variants or pseudogenes). It should be noted that Marangi et al. (20) analyzed members of haplogroup E and members of typical haplogroups B and C from samples in Italy, but the alignment of the sequences obtained in this study (region 2) with the GenBank sequences (region 1) was of poor quality due to poor management of large non-overlapping areas by the default program used (LR pers. obs.). Consequently, while nearly half of these new sequences belonged to *D. gallinae s.s*. as shown by Ciloglu et al. (24), they all formed a group together in Marangi et al. (20), creating an artifactual double clade in the position of a sister group to *D. gallinae s.s*. + *D. gallinae* L1.

The difficulties of morphological distinction reported between species of the *gallinae*-group (18) were confirmed, with only a few discriminating characters between *D. gallinae s.l., Dermanyssus carpathicus* Zeman, 1979*, Dermanyssus longipes* (Berlese and Trouessart, 1889)*, Dermanyssus hirundinis* (Hermann, 1804)*, D. apodis*, and many unstable characters within species (1). In particular, the distinction between *D. gallinae s.l*. and *D. apodis* is based on discriminant traits that are challenging for non-specialists to visualize (using a light microscope). Of course, the unavailability of morphologically discriminating characters within *D. gallinae s.l*. makes the morphological distinction between *D. gallinae s.s*. and *D. gallinae* L1 impossible (hence the term, cryptic species). However, since only *D. gallinae s.s*. has been found to be present in poultry farms in Europe and several parts of the world, the hematophagous mites to be distinguished in poultry farms in Europe and possibly in the world can be reduced to two very distant species *D. gallinae* (Dermanyssoidea: Dermanyssidae) and *Ornithonyssus sylviarum* (Canestrini & Fanzago, 1878) (Dermanyssoidea: Macronyssidae) (19). Therefore, the well-illustrated key proposed by Di Palma et al. (50) to distinguish *D. gallinae* from *O. sylviarum* is fully operational for studies restricted to layer farms, where *D. gallinae* is a problem. On the contrary, it may not be suitable for investigations in wild birds or even in other types of farming (pet bird farms, game farms, etc.).

### Integrative Analysis of Sequence Data

The sequencing of portions of the CO1 gene with different pairs of primers in the same mite individual in the present study (Tables 1, 2) did not reveal the presence of different copies, except for one individual (farm H). Identical copies were regularly amplified with different pairs since identical haplotypes are recurrently found in other studies. These elements make it possible to legitimize the comparison between sequences obtained in the different studies.

**Figure 3:**
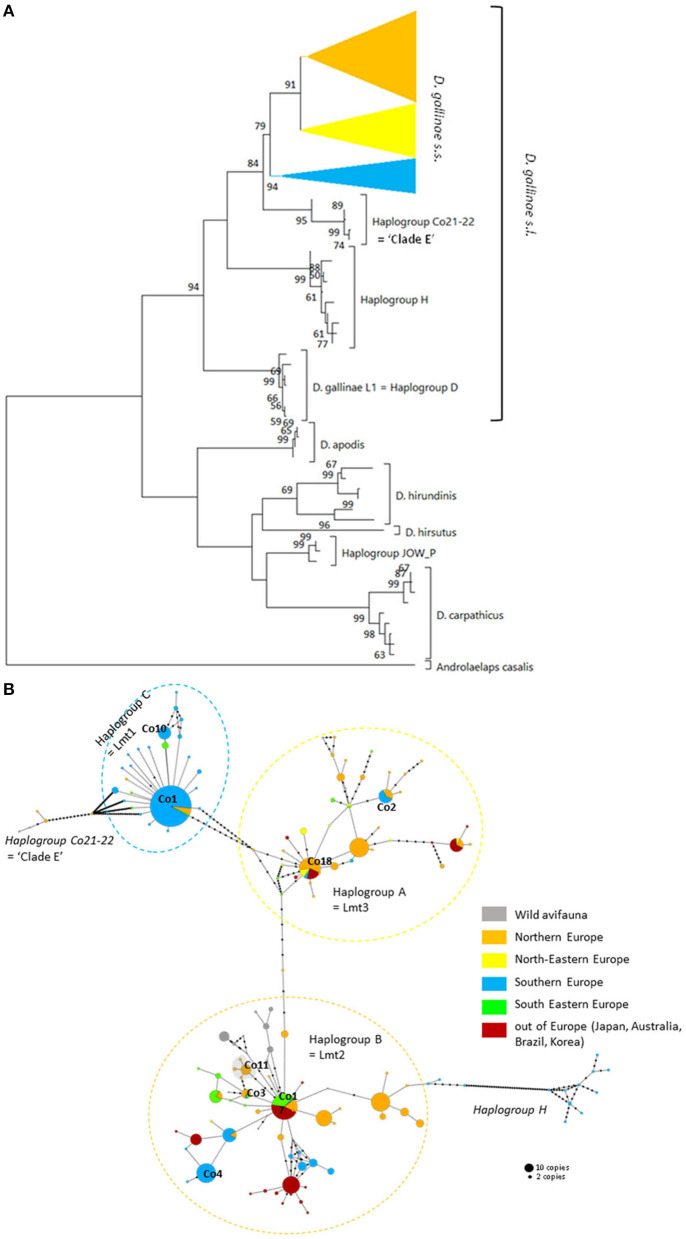
Genetic structure from CO1 haplotypes on region 1. **(A)** Evolutionary history of the different region-1 haplotypes obtained to date from the genus *Dermanyssus* with condensed clades within *D. gallinae s.s* (132 sequences) and *Androlaelaps casalis* (Mesostigmata: Dermanyssoidea) as an outgroup. A single copy of each haplotype has been integrated to the analysis (176 nucleotide sequences; alignment 470 bp long). It was inferred by using the Maximum Likelihood method and General Time Reversible model (51). The tree with the highest log likelihood (−4378.86) is shown. Initial tree(s) for the heuristic search were obtained automatically by applying Neighbor-Join and BioNJ algorithms to a matrix of pairwise distances estimated using the Maximum Composite Likelihood (MCL) approach, and then selecting the topology with superior log likelihood value. A discrete Gamma distribution was used to model evolutionary rate differences among sites (five categories; +G, parameter = 0.4639). The rate variation model allowed for some sites to be evolutionarily invariable ([+I], 25.74% sites). The tree is drawn to scale, with branch lengths measured in the number of substitutions per site. Bootstrap values >50% are displayed at the nodes. **(B)** Haplotype network (MSN) of the haplotypes in *D. gallinae s.s*. (*D. gallinae s.l*. excluding *D. gallinae* L1). Dashed lines delineate the three main haplogroups of *D. gallinae s.s*. recorded from region-1 data (the colors correspond to those of the condensed clades in **(A)**). The two atypical haplogroups in this dataset are indicated in italics. The size of the disks is proportional to the number of sequences present in the database and the IDs of some of the haplotypes isolated in (2) appear next to or on the disk. The four haplotypes in the shaded area including ‘Co11' are proper to the lab SK population. Geographical locations in Europe are as follows: Northern Europe (orange) includes Belgium, UK, Denmark, Norway, Sweden, Finland, The Netherlands; North-Eastern Europe (yellow) includes Poland and Czech Republic; Southern Europe (blue) includes France, Italy, Portugal; South Eastern Europe (green) includes Albania, Croatia, Slovenia, Turkey, Greece. Haplogroup C seems to be mainly present in Southern Europe, whilst Northern Europe and the present non-European countries are dominated with the haplogroups A and B. Black dots along the links between disks = mutations.

**Figure 4:**
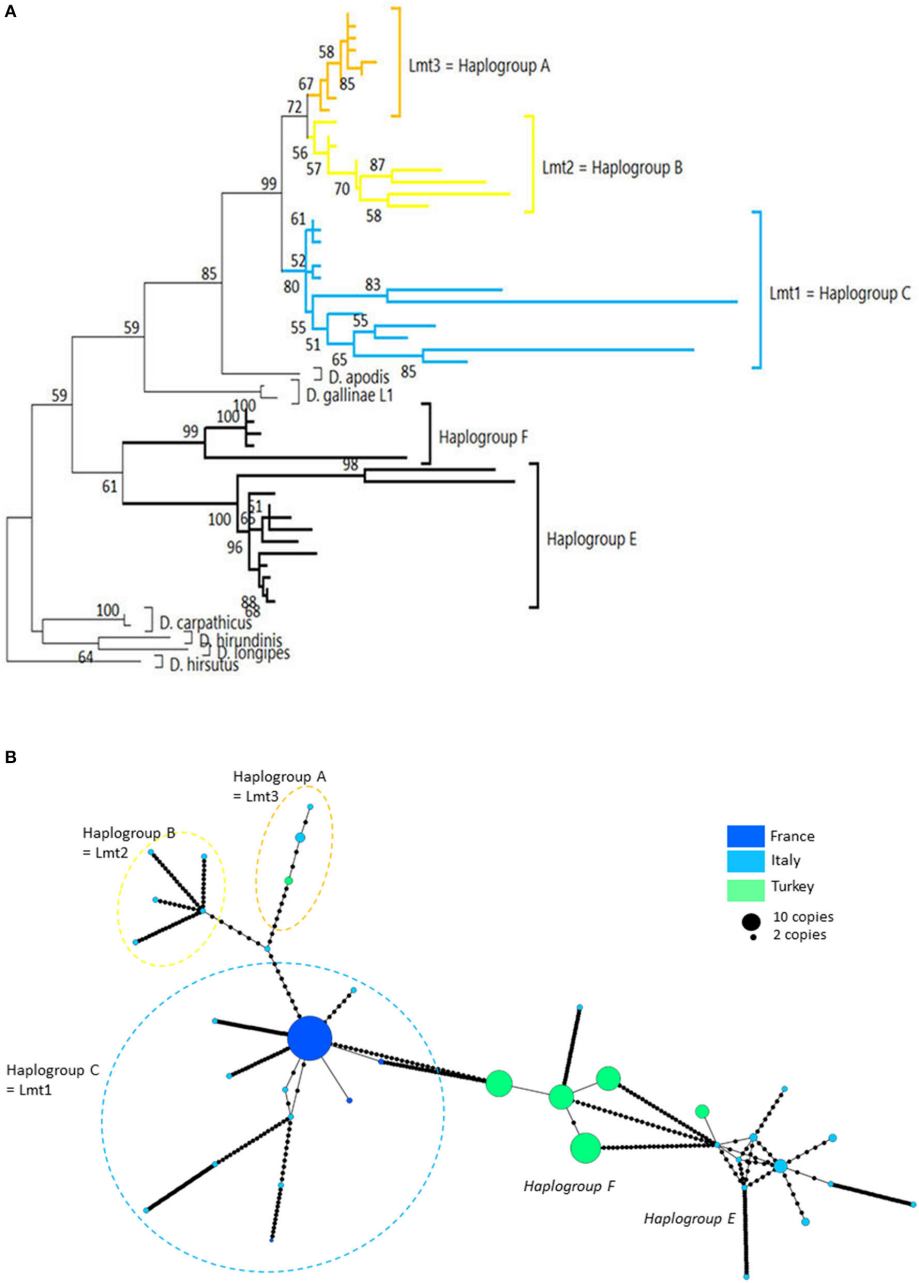
Genetic structure from CO1 haplotypes on region 2. **(A)** Evolutionary history of *Dermanyssus gallinae s.l*. CO1 haplotypes based on all available data on the region 2 of CO1 (273 bp) using the Tamura 3-parameter model (52), with sequences of other *Dermanyssus* species as outgroups (*D. apodis, D. carpathicus, D. hirundinis, D. longipes, D. hirsutus*). A single copy of each haplotype (55 nucleotide sequences) has been integrated to the analysis. The tree with the highest log likelihood (−2975.33) is shown. Only bootstrap values >50% are displayed at the nodes. In order to be able to root the tree with outgroups and to obtain a sufficiently informative topology, we have integrated sequences that are not completely overlapping into the analysis (2, 3, 20, 24), new data). The non-overlapping areas have been supplemented by *N* in the alignment. **(B)** Haplotype network (MSN) from CO1 region-2 data. In order to work on haplotypes of reasonable size that are completely resolved in this genomic region with some representation of the population structure, we have integrated into the analysis exclusively the data from (20, 24) (Turkey) and the present data obtained for region 2 (four populations; France). Dashed lines delineate the only typical haplogroups of *D. gallinae s.s*. recorded from region 2 data (the colors correspond to those of the condensed clades in **(A)**). The two atypical haplogroups in this dataset are indicated in italics. The size of the disks is proportional to the number of sequences present in the database and the name of one haplotypes identified in (2) appears on the disk (‘Co1'). Black dots = mutations.

Three mitochondrial lineages known since 2011 are designated by two series of identifiers (mitochondrial lineage Lmt*n* or haplogroup *N*) that revealed to be exactly synonymous (Figures 3, 4; Table 4). The mitochondrial lineages Lmt1, Lmt2, and Lmt3 determined by phylogenetic reconstruction by Roy et al. (2) and defined as second-level clusters by Bayesian clustering by Roy & Buronfosse (4) match exactly the haplogroups C, B, and A defined by Øines & Brännström (3) by phylogenetic reconstruction and taken up by Chu et al. (22) and Ciloglu et al. (24) (Supplementary Material 1). These three lineages, widely represented in the different studies, will be referred to in the rest of the text as “typical haplogroups” of *D. gallinae s.s*. and the letters A, B, and C will be used to name them.

**Figure 5:**
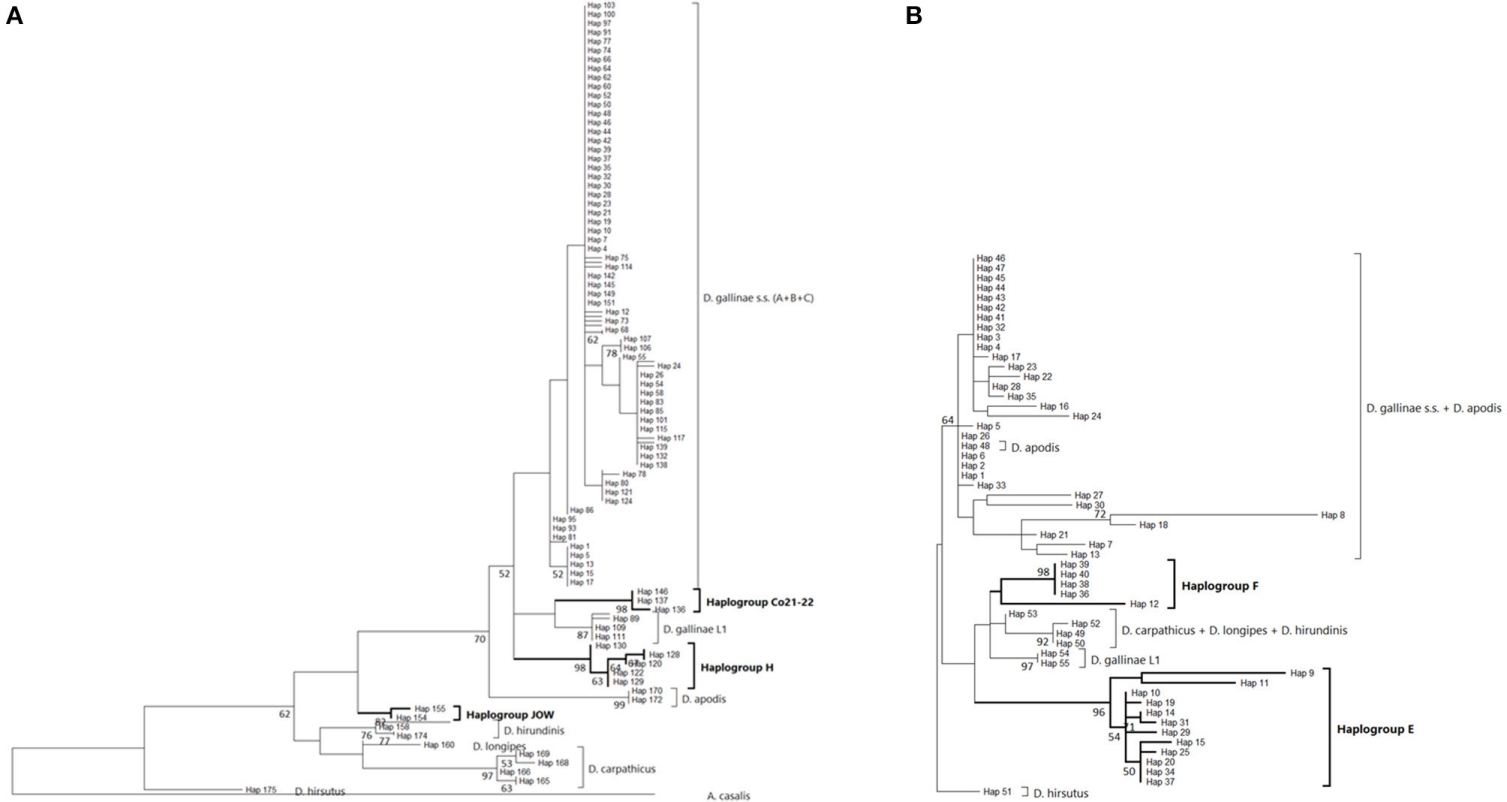
Evolutionary history of *Dermanyssus gallinae s.l*. CO1 haplotypes based on 1st codon positions of available CO1 data using the Maximum Likelihood method. A single copy of each haplotype has been integrated to the analyses. Only bootstrap values >50% are displayed at the nodes. In bold, atypical haplogroups. **(A)** Region-1 dataset (176 nucleotide sequences and 157 positions). The General Time Reversible model (51) was used with a discrete Gamma distribution to model evolutionary rate differences among sites (five categories (+*G*, parameter = 0.4599) and the rate variation model allowed for some sites to be evolutionarily invariable ([+*I*], 31.21% sites). The tree with the highest log likelihood (-813.96) is shown. **(B)** Region-2 dataset (55 nucleotide sequences and 91 positions). The Tamura-Nei model (53) was used with the rate variation model allowed for some sites to be evolutionarily invariable ([+*I*], 17.58% sites). The tree with the highest log likelihood (−737.98) is shown. Note: the longest branches in the *D. gallinae s.s*. + *D. apodis* clade are due to sparse differences in the haplotypes belonging to the typical haplogroups from Marangi et al. (20), likely due to double sequences (haplogroup E + typical haplotypes).

Four atypical haplogroups in mites morphologically conforming to *D. gallinae s.l*. have been reported in the literature, namely JOW in (19), ‘Co21-22' in (2), haplogroups E and F in (24) (see above), and a fifth appears in the new data, namely haplogroup H. Of these, four have been encountered in poultry farms: JOW (in the USA), E and F (in Italy and Turkey), H (in France). In contrast to the three typical poultry and cosmopolitan haplogroups detailed above, at least the last three (E, F, and H) show a much narrower distribution, as they have only been found occasionally in regions of Europe that have been explored in detail (Turkey, Italy) and where typical haplotypes are encountered recurrently [see (23) and Figure 3]. Predictive translation of the nucleotide sequence revealed that the sequences of haplogroup H contain an indel followed by three stop codons. The other CO1 sequences (region 1 and region 2) do not contain any other stop codon; however, substantial differences in the aminoacid sequences translated from the obtained DNA sequences exist between the atypical haplogroups E and F on the one hand and the whole *Dermanyssus* sequences, including *D. gallinae s.l.*, and the outgroups on the other hand (Supplementary Material 2). Considering all positions of the nucleotide sequences, these atypical haplogroups, although, carried by mites morphologically conforming to *D. gallinae s.s*., were located outside this species, amongst the outgroup species of the genus *Dermanyssus*, according to the phylogenetic ML inference: with the region 1 dataset, the JOW haplogroup was located in the middle of the clades of outgroups (Figures 3, 4) and with the region 2 dataset the atypical haplogroups E and F form a sister clade to *D. gallinae s.l*. + *D. apodis*. Considering only the first position of the codons, the resolution of typical haplogroups decreased drastically in accordance with what is expected for a functional genomic region, and the resolution of the three atypical haplogroups remained high, with long and strongly supported branches (Figure 5). Lastly, Tpm sequences obtained from mites carrying the haplogroup H share Tpm alleles of mites carrying typical haplogroups of *D. gallinae s.s*. (Supplementary Material 3).

## Discussion

### Typical Mitochondrial Haplogroups A, B, C and Signs of Expansion Within *D. gallinae* s.s.

**Figure 6:**
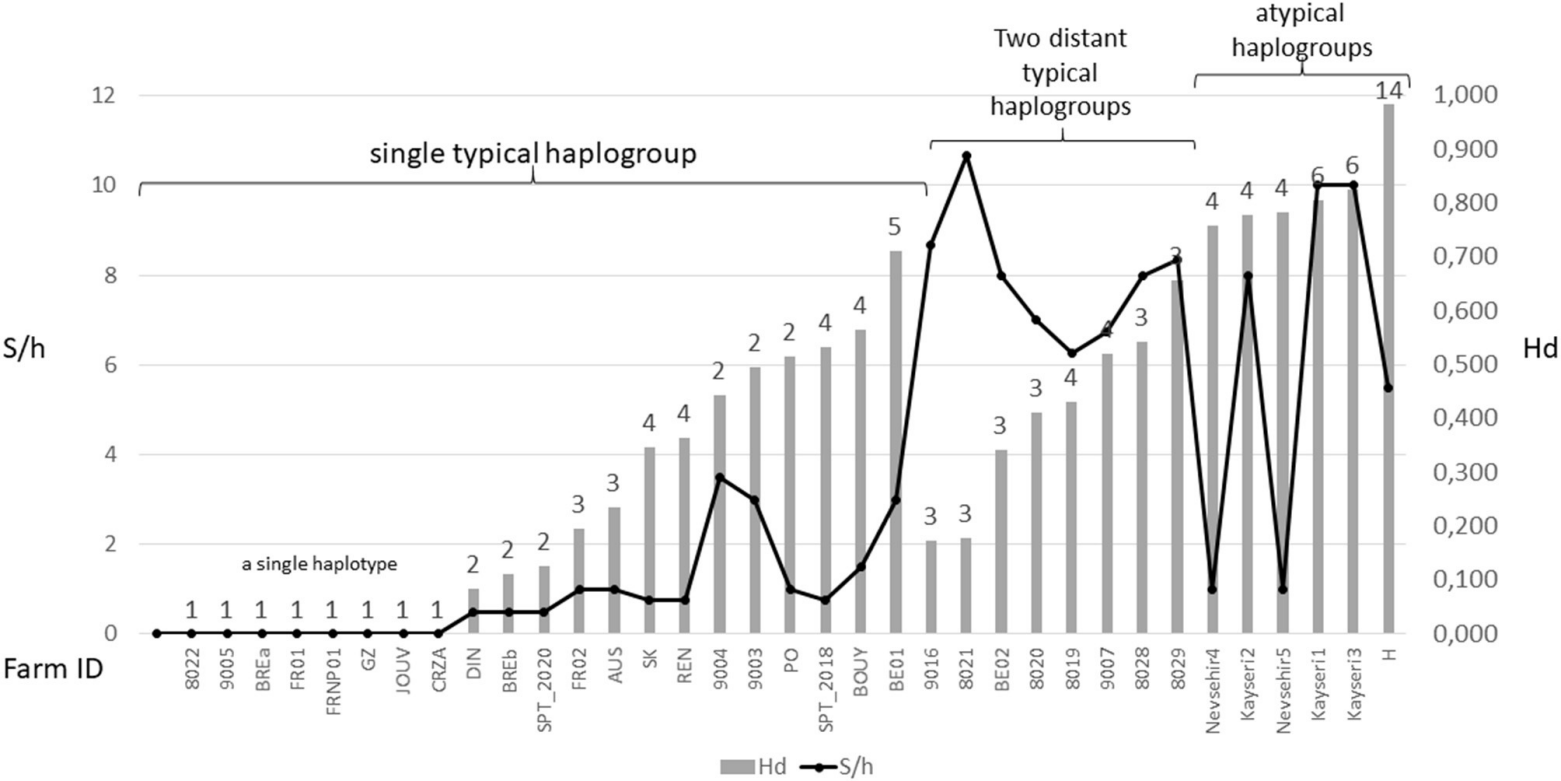
Overview of diversity indices based on CO1 haplotypes in *D. gallinae s.s*. populations encountered in farms. The numbers above the bars represent the number of haplotypes encountered in each farm sample. Hd, haplotype diversity; S/h, ratio of the number of segregating sites to the number of haplotypes detected in an isolate. Values are ordered from left to right within three different mt profiles: populations carrying a single typical haplogroup, populations carrying two or three typical haplogroups including C and populations carrying any atypical haplogroups. The data set is presented in Supplementary Material 3. High S/h values were always recorded in farms where haplogroup C and one or two of the other haplogroups were found together, regardless of the Hd value. High Hd values (>0.75) were found in farms where atypical haplotypes have been amplified (haplogroups E, F, and H), likely due to the rapid evolution of the non-functional sequences.

The three typical haplogroups show recurrent signs of expansion: several star patterns in haplotype networks are formed by multiple rare variants very closely related (1–2 mutations) to a strongly represented central haplotype [in particular haplotypes respectively named ‘Co1,' ‘Co17,' ‘Co18' in (2, 4) and Figure 3B]. This is consistent with large populations that develop rapidly from a small number of individuals after undergoing a drastic reduction (bottleneck events) or after a small number of individuals have colonized a new area (founder events), thus growing from only a few haplotypes if not a single one. The former may typically happen after a massive acaricide treatment and the latter after contamination of an uninfested farm. Over many generations, if kept isolated, such a population will only contain the initial haplotype(s) and a few closely related recent variants, i.e., haplotypes emerging by random point mutation within the population. The low local variety of haplotypes supports this hypothesis, with almost always a single haplotype being the dominant haplotype within a poultry house or group of nests [(3, 4, 22), new data, Figure 6]. This type of process well explains the three-point star-like structure formed in the CO1 haplotype network by haplotypes isolated from the laboratory population SK (‘Co11', Figure 3). It was observed that after mites were collected from a Danish layer farm, this SK-population underwent a marked bottleneck event during the initiation of its culture in the laboratory (Kilpinen, pers. comm.). This population was then grown in the laboratory for more than a decade (between 1997 and 2009), maintained in inbreeding (without adding new mites) over >430 generations (assuming three generations per month). During this period, it seems like three new haplotypes appeared, differing by one nucleotide each from the majority one, as a result of three independent mutation events. The probability that such an inheritable mutation (a random event during gamete formation) will appear in the gene region under consideration is naturally increased by the population's size. The much larger population size on the scale of interconnected farms compared to a laboratory facility explains well the much higher number of closely related variants (stars' points) of the haplotypes ‘Co1,' ‘Co17,' ‘Co18.' Oh et al. (21) detected exclusively the ‘Co18' haplotype in 13 Korean farm buildings, which might suggest a very recent invasion in these farms. However, as these authors carried out only one sequencing per building, the rare recent variants may have been missed.

Although, the typical composition of a farm population is centered around a single major haplotype with a small number of close variants, in some cases, two or even three typical haplogroups have been encountered at the same time in single laying hen facilities (4, 23, Øines pers. comm., present study). From a diagnostic point of view, this substantially increases the number of segregating sites per haplotype S/h within the poultry house, without necessarily increasing the haplotypic Hd diversity (Figure 6). This pattern can be explained by a new contamination event in an already infested farm, producing secondary contact between long separated populations (see below).

### Atypical Haplogroups E, F, H = NUMTs Pseudogenes

From *in-silico* analysis of the sequences in the JOW, CO21-22, E, F, and H haplogroups, we can conclude that these are clearly non-functional given the important differences on positions 1 and 2 of the codons, including the presence of one stop codon, in the haplogroup H. While the existence of stop codons in a protein-coding sequence is a strong indication of loss of functionality, their absence (as in JOW, CO21-22, E, and F) is not evidence of functionality (54). On the other hand, the persistence of the strongly supported clades of atypical haplogroups, while those of typical haplogroups disappear when the third positions are omitted (comb-like; Figure 6), supports the loss of functionality in the former. Since most of the synonymous nucleotide substitutions (which do not produce an amino acid change, therefore more likely to be a substitution which does not affect the fitness of the organism) are on position 3, it is consistent to no longer perceive differentiation when neglecting position 3 of functional sequences of closely related organisms. Conversely, the maintenance of groups formed by other sequences is explained by the relaxing of the selection pressure following the loss of their functionality. At higher taxonomic levels, non-silent substitutions can, however, provide valuable information on phylogenetic relationships between distantly-related organisms: removing the third position can help address compositional heterogeneity and saturation, which may occur due to frequent changes in the third codon position in datasets of CO1 from organisms whose divergence is ancient and can lead to long-branch attraction (55). The 4 NUMT lines found in the present study seem to originate from different ancestral haplotypes, which suggests that several events of incorporation into the nuclear genome of *D. gallinae* may have occurred independently, as has been reported in some insects (56). Lastly, the pseudogenic status is corroborated by the absence of evidence of advanced speciation in the Tpm nuclear DNA (alleles are shared between atypical-H mites and typical-A-B-C mites). For comparison, the reproductive isolation of *D. gallinae* L1 is supported both by a long and strongly supported mitochondrial branch (Figures 3, 4) and by original nuclear Tpm alleles, i.e., not shared with any of the mites carrying typical *D. gallinae s.s*. haplotypes (Supplementary Materials 3, 4). All these sequence characteristics of stop-free atypical haplogroups strongly suggest that they are also NUMTs, even if the relaxation of the selection pressure has not yet resulted in the occurrence in the present sample of stop codons in all of them.

At the population level, the frequency and spatial distribution patterns of atypical haplogroups further consolidate this hypothesis. The atypical haplotypes encountered in the farms sampled at population level by (24) (E, F) and in the present study (H) show very different demographic profiles from typical haplotypes: several related variants are present within the same henhouse with relatively balanced frequencies (absence of star pattern, Figures 3, 4; higher Hd value than in farms without atypical haplogroup, Figure 6). The rapid evolution of non-functional sequences probably explains this scattered pattern different from the expansion patterns found in typical haplogroups. For the atypical haplogroups JOW and Co21-22 (clade E of (1) + new “Quail” isolate), the limited sampling does not provide an accurate representation of the population and thus demographic profiles. However, the fact that 3 different haplotypes were found among 4 individuals in the new “Quail” isolate and the JOW isolate suggests a similar distribution pattern to the other atypical haplotypes.

Interestingly, all the sequences provided by (20), which were not linked to haplotype E and were assigned here to the typical haplogroups A, B, and C show a clear excess of non-synonymous mutations when considering the corresponding amino acid sequences (Supplementary Material 2). This excess translates into excessively long branches and links in the phylogenetic tree (Figure 4A) and the network of haplotypes (Italy in Figure 4B), respectively. This could be explained by double peaks in the chromatograms, generated by the simultaneous amplification of a NUMT with its mitochondrial counterpart, as was observed in the present study with mites from the H isolate (six individuals out of 20 discarded from the analysis because of too poor quality chromatograms due to the presence of a multitude of double peaks). Here, the absence of indels makes it impossible to deduce the two sequences by Indelligent analysis (search for the repetition of sequence portions with a shift of the length of the indel) or internal sequencing as was done for Tpm (4). This is why we warn prospective researchers against using such chromatograms for future mite studies.

### Ecological and Geographical Distribution of the Genetic Groups of *D. gallinae s.l*.

*Dermanyssus gallinae s.s*. has a much broader host range than other species within *Dermanyssus*. It is the only species encountered in birds belonging to different taxonomic orders [at least nine orders according to (28)]. It is also the only species encountered in poultry farms to date. All species of *Dermanyssus* other than *D. gallinae s.s*. have been recorded in a single family or genus of bird or a small number of species of the same order nesting in the same environments and conditions (28). The cryptic *D. gallinae* L1, although morphologically indistinguishable from *D. gallinae s.s*., also has a narrow host range: it was found almost exclusively in pigeons, both in wild (urban) nests and pigeon farms, in both Europe and the USA. Many morphology-based studies have shown that mites belonging to *D. gallinae s.l*. also occasionally bites humans [see (57) for review]. Amongst them, in the few morpho-molecular studies published so far on human cases, *D. gallinae* L1 has been shown to cause human disease in urban areas on at least two occasions (25, 27). Conversely, a haplotype of *D. gallinae s.s*. typical of layer farms has been reported in humans in a third case report (26). The One Health potential of this mite should not be ignored, not only as being the cause of human gamasoidosis, but perhaps more importantly, with an unknown impact by its vector capacity, for poultry and humans as several bacterial and viral pathogens has been described from it.

In poultry farms, the three typical haplogroups of *D. gallinae s.s*. are widely represented in Europe, as well as in the other regions of the world studied so far [Japan (22), Korea (21), Brazil, and Australia (4)]. This wide distribution is almost exclusively related to human activities: populations that develop in wild avifauna have been shown to be highly isolated reproductively (3, 4). However, a geographical structure is emerging since haplogroup C is present mainly in southern European countries and haplogroup B in northern European countries (Figure 3B). Haplogroup A is the most cosmopolitan. Within the three typical haplogroups of *D. gallinae s.s*., Øines & Brännström (3) detected signs of differentiation between populations collected from Swedish and Norwegian farms consistent with the administrative functioning of these two Scandinavian countries: series of closely related haplotypes are present in both countries, but none are shared.

On the other hand, Swedish farms share haplotypes with the rest of Europe. This is consistent with foreign trade being much more developed in Sweden as it is part of the European Union, where there is an open policy of movement of goods and services, while Norway not being part of the EU. The tariff protection on meat and egg products between Norway and its European neighbors ensures a good domestic market for Norwegian domestic production of agricultural products, implemented by the Norwegian government (58). This legislation helps control the import of live chickens to reduce the import of poultry diseases (59). With a generally increased burden of bureaucracy and documentation need when importing animals, this is likely to limit the flow of potentially contaminated products or animals. This trade barrier may have contributed to Norway having one of the lowest rates of mite-infected layers in Europe (60).

**Figure 7:**
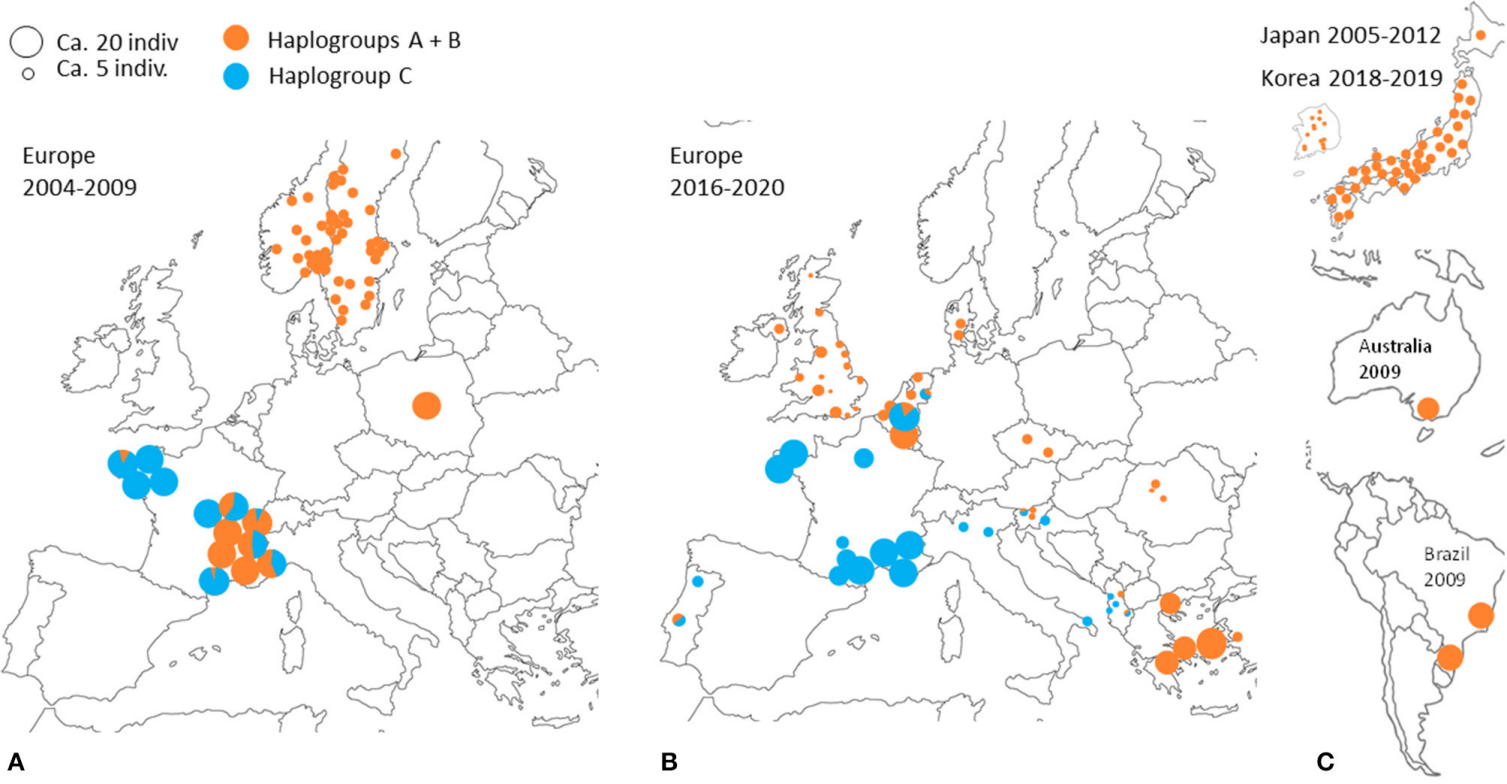
Geographical distribution of typical CO1 haplotypes A+B and C at different periods according to data from (3, 4, 21–23). A disc represents a set of mite individuals collected from the same poultry house (or sometimes from several poultry houses within the same geographical entity) and the colors indicate the proportion of individuals carrying haplotypes A or B (orange) and individuals carrying haplotypes C (blue). The size of the disc is proportional to the number of individuals sequenced. **(A)** Previous decade in Europe (2004 and 2009). **(B)** Current decade in Europe (2017–2020). **(C)** Previous decade and the beginning of the current decade in other regions of the world.

Despite the motley and irregular nature of the available sampling, the accumulation of information on the European distribution of haplogroups of *D. gallinae s.s*. by successive studies and our current data shows variations in time and space consistent with a massive expansion process in Europe of haplogroup C from a point of origin in or near France (Figure 7). A major socio-economic reorganization of the poultry industry around Lyon in France late 1990's might have generated the mixing between two strongly differentiated genetic groups (haplogroup C vs. haplogroups A+B, A and B being more closely related to each other): the distance traveled by trucks transporting spent hens was abruptly increased as a result of a shift from a configuration with multiple local slaughterhouses to an arrangement with large centralized slaughterhouses in regions far from Lyon, particularly in Brittany (and outside France; Lubac, ITAVI, pers. comm.). Ca. 10 years later, Roy & Buronfosse (4) observed in 2008–2009 several populations with a mixed mitochondrial profile in these two distant French regions. The inverted proportions of haplogroup C vs. A+B (haplogroup C majority in Brittany (NW France) and haplogroups A and/or B globally majority around Lyon (SE France); Figure 7) suggested a recent transfer of haplogroup C from Brittany to Lyon's region. During the last decade, haplogroup C seems to have expanded from Brittany to the SE zone of France via poultry transport within France. During the current decade, haplogroup C remains concentrated in France, but is also found in surrounding countries (Portugal, Italy, Belgium, the Netherlands).

Interestingly, the other typical haplogroups have not been found in France during the last decade, whereas they were frequent in the previous decade sampling. The disappearance of the other haplogroups in samples from France in the current decade suggests an intraspecies competitive exclusion effect or particularities favoring the persistence of haplogroup C in hybrid populations [cytoplasmic incompatibility due to a bacterium, for example; (61)]. The concomitant presence of this haplogroup and another typical haplogroup in a Belgian henhouse, as well as in four other sampling points in Portugal, the Netherlands, Slovenia and Albania, suggests that a process of geographic expansion is underway in the countries around the probable country of origin: new contamination events seem to bring haplogroup C into contact with other haplogroups in France's neighboring countries. This apparent expansion is consistent with the demographic profile of expansion observed around the ‘Co1' haplotype in the CO1 network of Figures 3, 4 and is consistent with its relatively recent local origin in France.

## Conclusions, Perspectives, and Recommendations for Future Studies

This study provides a framework for future studies requiring knowledge about interspecific boundaries (who is who?) and spread routes (who goes where?) of PRM. Layer farms in the parts of the world studied so far are infested by three mitochondrial haplogroups (A, B, C), one of which appears to be expanding (C). It would be interesting to explicitly test the hypothesis of an ongoing invasion by this haplogroup and to study the effects of hybridization between haplogroups A, B, and C. Indeed, if, as suggested by the spatio-temporal distribution of the haplogroups in the available data, the genetic structure of the populations infesting the farms in the northern parts of Europe is indeed being modified, changes in the response to treatments or the effect on the animals could take place in the coming years depending on the respective characteristics of these genetic groups and the effects of their possible hybridization. To anticipate any possible changes in the behavior of *D. gallinae s.s*. infestations, it would therefore also be advisable to verify that these haplogroups are indeed interfecund and to study the differences in treatment resistance, pathogenicity and/or vector capacity between haplogroups and/or between hybridized and not hybridized populations.

Our study also points out that it is possible that NUMTs can confound the analysis and methodological aspects when studying variations in mitochondrial sequences in *D. gallinae* and calls for the researcher to be vigilant and perform necessary QC actions on the data. The absence of stop codons may not be sufficient to ensure that the amplified sequences are, in fact the targeted mitochondrial sequences when working with the gene encoding CO1. It is recommended to sequence individuals separately, perform this on several individuals per henhouse, and to consider several criteria to avoid pollution of the analysis by NUMTs, especially when working on region 2 as our analysis indicate it is the portion of CO1 in which the most significant proportion of individuals have resulted in the sequencing of NUMTs so far. In case of divergence from the CO1 sequences of the three mitochondrial haplogroups typical of *D. gallinae s.s*. (or *D. gallinae* L1) in mites whose morphology conforms to *D. gallinae* according to (1), or simply according to (50) in the case of sampling from layer farms, the following indicators constitute warnings:

1. Amino acid CO1 sequence showing several segregating differences (within the sample) with respect to the sequences of *D. gallinae* s. l. individuals available in the GenBank and/or the maintained resolution of the new grouping in phylogenetic analyses conducted on the first codon positions only (whereas, the groupings of A, B, and C are lost).
2. Discrepancies between CO1-based phylogenetic trees and the taxonomic framework. Integrating the sequences of several species of the genus *Dermanyssus* (if possible even several individuals per species; to be collected from the GenBank) into the phylogenetic analysis allows to visualize the positioning of the tested sequences in relation to that of the other species and to detect possible discrepancies such as those found with the haplogroup JOW.
3. Multiple related CO1 haplotypes within a building with a more or less balanced frequency of each (Hd value >0.70). This feature requires a quite intensive sampling effort (ca. 20 individuals per henhouse) to be assessable.
4. Careful examination of chromatograms of CO1 sequences containing series of polymorphic sites and double peaks. Care when using cloning methods in conjunction with sequencing as a wrong copy may be characterized. This may result from concomitant amplification of both the functional mitochondrial and NUMT sequences if performing PCR on DNA from a single mite.
5. Nuclear alleles of variable regions (e.g., Tpm, microsatellites.) shared with mites carrying typical haplotypes.
6. We warmly encourage those planning to conduct further studies on the genetic structure of *D. gallinae* to check at least warnings #1 to #4, and ideally, in case of doubt, to supplement the analysis with nuclear markers (warning #5). In addition, it is recommended to work preferentially on carefully preserved mites because it has been found in other arthropods that NUMTs were more often amplified from long-dried individuals (14). Zhang et al. (62) noticed that in some dried insects, the nuclear sequences were preferentially amplified instead of their mitochondrial counterpart. Therefore, these authors and Leite (14) recommend to work if possible on fresh specimens or at least on well-preserved ones. Ideally, mites are placed alive at −20°C after a few days at RT° to allow blood digestion (since blood contains esterases that can disrupt PCR amplification) either dry or in ethanol at >95°C.

In addition, given that gamasidosis is becoming a growing One-Health problem for both humans and other mammals (57, 60), that mites associated with pigeons or hens have been identified as the culprits, and that the distinction between *D. gallinae s.s*. and *D. gallinae* L1 can only be made on a molecular basis, the definition of a PCR diagnostic protocol in human and veterinary medicine could prove useful to enable practicing veterinarians, physicians, dermatologists, acarologists, to identify the source of human gamasidosis clearly.

Finally, based on the present state of the knowledge acquired using the Sanger sequencing method, several promising research directions can be taken by capitalizing on two major advances:

- Complete genomes of a few *D. gallinae* isolates, one nuclear and one mitochondrial, have recently been released in public databases: GenBank assembly accessions GCA_003439945.1 [nuclear genome, (63)], MW044618.1 (complete mitochondrial genome).
- High-throughput sequencing technologies are improving substantially while becoming increasingly affordable.

The availability of the complete nuclear genome of PRM opens major prospects for the development of microsatellite and/or SNP markers, rapidly evolving markers more appropriate than nuclear Sanger sequences for intraspecific analysis thanks to analytical methods allowing easy management of heterozygotes. In addition, while barcoding allows rapid assignment of individuals to species in relatively well-studied taxonomic groups based on a single genomic region, i.e., a single locus, relying on one or two loci to determine the neutral genetic structure of populations within the species and to analyze spread routes has significant drawbacks. Even if the said markers (here CO1 and Tpm intron) extend over several hundred nucleotides, have high intraspecific variability and are likely to be subject to little selection effect, these are in the end single loci, the different nucleotidic sites being strongly linked to each another. There is, therefore a significant risk that their variation does not provide sufficiently reliable evidence of the evolutionary history of the genomes of which they constitute a very small sample. The heterogeneity of the differentiation of the different loci within the genome between populations is now known, resulting from the contrasting effects of natural selection (divergent differentiation), gene flow (homogenization) and variations in mutation rates across the genome [see (64)]. Thanks to genomic data, one can now easily design a few dozen microsatellite markers or SNPs randomly distributed in the genome of *D. gallinae* and compare multilocus genotypes from different populations. This is one more reason to consider that future microsatellite markers and/or SNPs will bring a lot.

High-throughput sequencing technologies that are becoming increasingly affordable (e.g., Illumina sequencing to obtain many short sequences or nanopore sequencing to obtain very fast long sequences) will allow even more rapid development of multilocus markers, using, for instance, restriction site-associated DNA sequencing [RAD-seq; (65)]. These advances should allow us to refine our understanding of the expansion process of haplogroup C and consequently develop relevant recommendations to improve prophylaxis in farms in different European countries. However, when using NGS approaches, there should be care taken on the dataset, especially when analyzing batches made up of DNA from several individuals simultaneously, as pseudogens may be encountered in this material, and results could wrongfully give indications of the presence of new haplogroups. In addition, high-throughput technologies will also make it possible to carry out studies of population genomics crossing phenotypes and genotypes using genome-wide QTL (quantitative trait locus) mapping. For example, Bulked Segregant Analysis (BSA) on susceptible and resistant populations crossed in the laboratory under controlled acaricidal pressures could allow the identification of mutations responsible for resistance, as has been done on *Tetranychus urticae*, a plant mite where polygenic resistance to METI (Mitochondrial Electron Transport Inhibitors) has been demonstrated (66).

## Data Availability Statement

The datasets presented in this study can be found in the GenBank repository. The accession number(s) are MW392987-MW393187 (mt-CO1), MW401582-MW401598 (CO1-NUMTs), MW401552-MW401581 (Tpm intron). Detailed correspondances between accession numbers, DNA region, mite individuals and sampling location can be found in Supplementary Data Sheet 1.

## Author Contributions

LR and ØØ designed the project with contribution from AG. LR conducted the analyses and wrote the first version of the manuscript. LR and NS obtained the funding. All authors contributed to the article and approved the submitted version.

## Conflict of Interest

The authors declare that the research was conducted in the absence of any commercial or financial relationships that could be construed as a potential conflict of interest. The reviewer AN declared a past co-authorship with the authors LR and ØØ to the handling editor.

## Footnotes

^1^This is contrary to what is known in other organisms, but consistent with what has been previously noted in other acari (38–40). This defect was confirmed in (3) and (22). These authors reported that the substantial variety of CO1 haplotypes found in hundreds of farms was accompanied by a negligible variation among ITS sequences (1 to 2 sequences max, differing by <0.4%). It is therefore unnecessary to use ITS for intraspecies investigation in *D. gallinae*.

## Supplementary Material

Supplementary Material 1 Distribution of the groupings assigned by the authors of the studies considered here in the evolutionary history of the different CO1 haplotypes in *D. gallinae*.

Supplementary Material 2 Amino-acid sequences translated from the CO1 haplotypes (a single copy per haplotype) of *D. gallinae*. The sequences of haplogroups H (region 1 data set; left) and *E and F (region 2 data set, right) show substantial differences from the sequences of D*. *gallinae s.s*. and other *Dermanyssus* species. The differences are more important between these haplogroups and the non-*gallinae* species. Sequences from the (20) dataset related to typical haplogroups of *D. gallinae s.s*. (A, B, C) show scattered differences in amino acid sequence, probably due to the concomitant amplification of a NUMT and a true mitochondrial sequence. The CO21-22 and JOW haplogroups also show some differences in the composition of their sequences compared to the others, but this is less marked.

Supplementary Material 3 Heatmap of the frequencies of Co1 haplogroups and Tpm alleles within the isolates studied by (4, 24) and in the present study, as well as diversity indices. No clear association between a mitochondrial lineage and a nuclear allele appears within *D. gallinae* s.s. (all Tpm alleles are present with substantial frequencies in any of the isolates attached to each haplogroup). On the other hand, there is a marked separation between the two wildlife populations and all other populations.

Supplementary Material 4 Evolutionary history of Tpm alleles (nuclear DNA) recorded from *Dermanyssus* until now, ie from (2, 4) and the homozygous sequences from present data (1,026 nucleotide sequences; total of 735 positions in the final dataset). On farms, most heterozygous individuals carry two distant alleles, belonging respectively to the clades Tro 1, Tro 2, or Tro 3.

Supplementary Data Sheet 1 Sampling information associated with accession numbers of DNA sequences were obtained in the present study.
