# Supplementary material for: Who Is Dermanyssus gallinae? Genetic Structure of Populations and Critical Synthesis of the Current Knowledge
Source: Front Vet Sci. 2021 May 28;8:650546. doi: 10.3389/fvets.2021.650546 (PMC8194076; doi:10.3389/fvets.2021.650546)
Supplement: Supplementary Material 1 — Distribution of the groupings assigned by the authors of the studies considered here in the evolutionary history of the different CO1 haplotypes in D. gallinae. [file Presentation_1.pdf]

Supplementary material 1. Distribution of the different groupings assigned by the authors of the studies considered here in the evolutionary history of the Co1 haplotypes in *D. gallinae.*

For each haplotype for which information was available in the study or in the studies where its sequence was used, an indication is placed on the right, as follows: in order, second level cluster according to Roy & Buronfosse (2011 (4); Lmt1, Lmt2 or Lmt3), haplotype according to Roy et al. (2010;(2)) in brackets ("Co#"), haplogroup A, B, C according to Oines & Brännström (2011; OB (3)) or to Chu et al. (2016; CHU (22)) or to Ciloglu et al. (2020; CIL (24)). A. Region 1 (subtree of *D. gallinae* s.s. from tree in Fig.2). B. Region 2 (full tree).

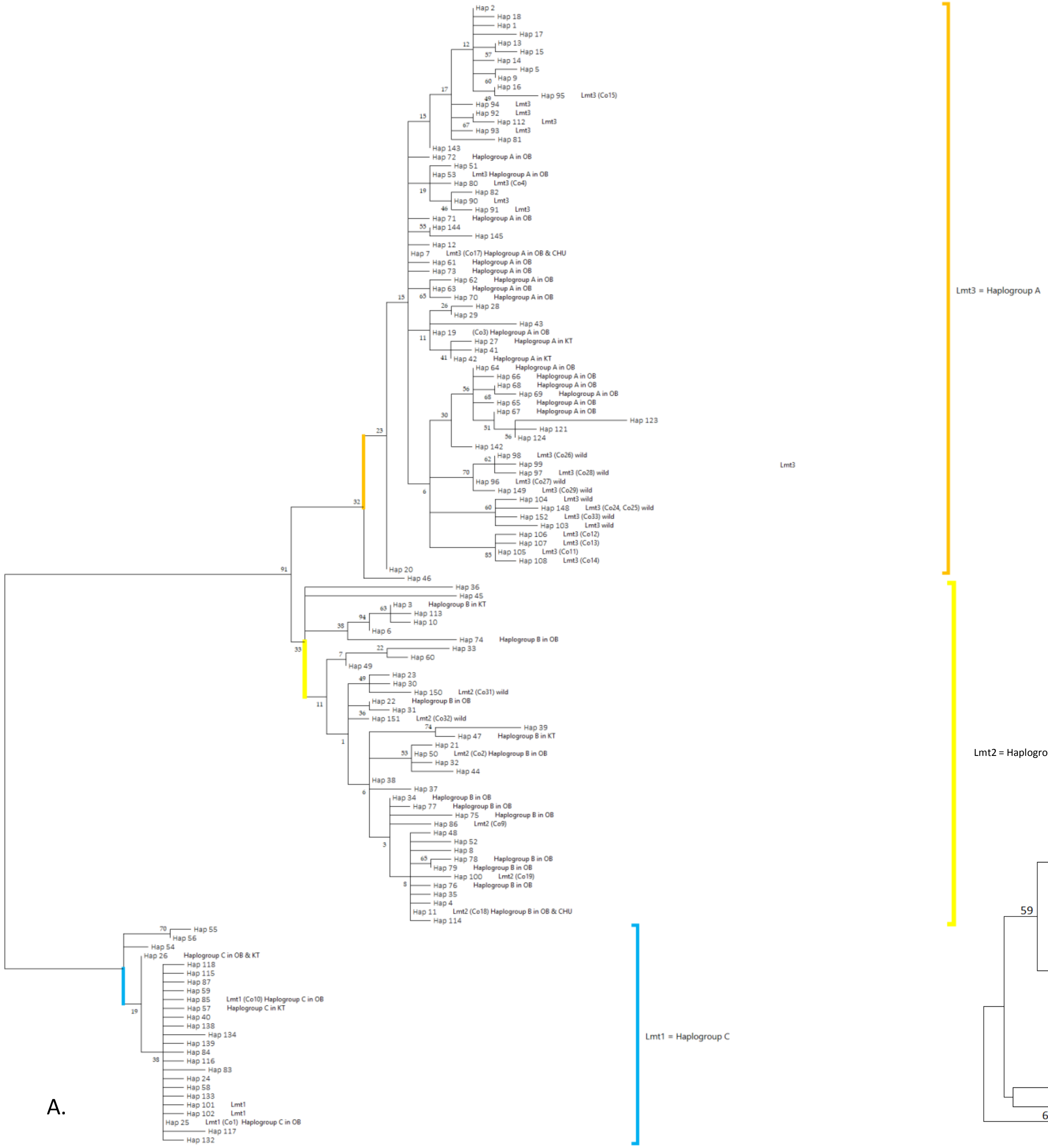

A.

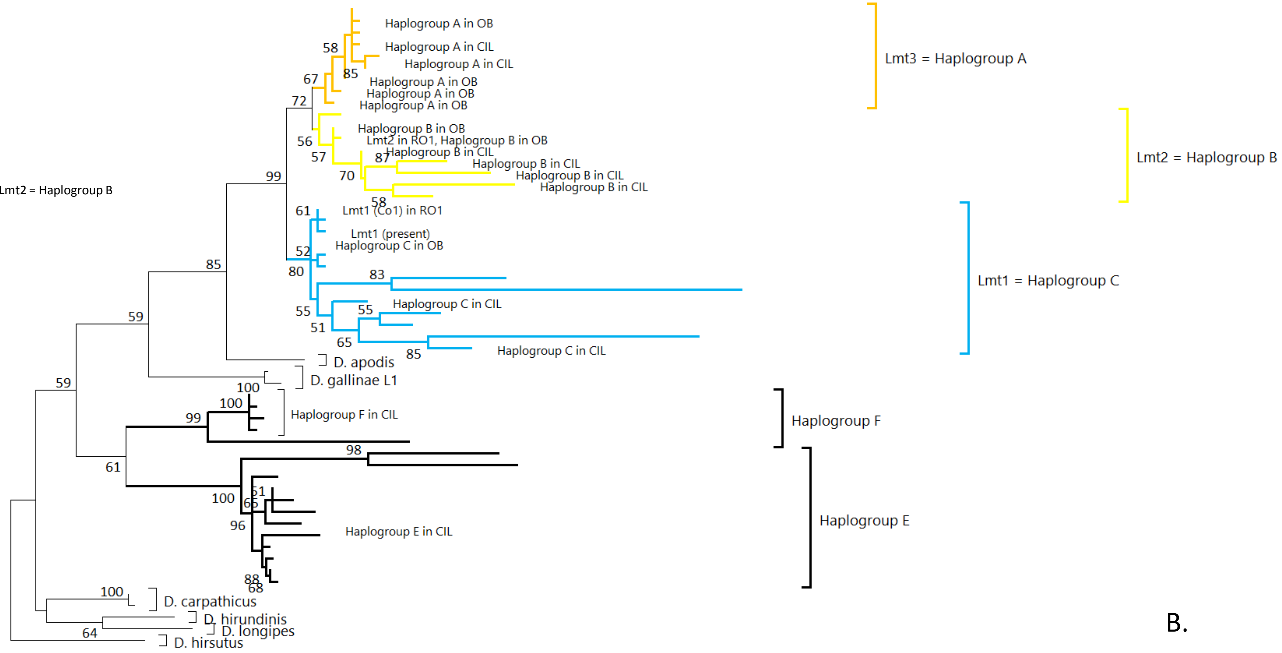

B.

Supplementary material 2. Amino-acid sequences translated from the Co1 haplotypes (a single copy per haplotype) of *D. gallinae.*

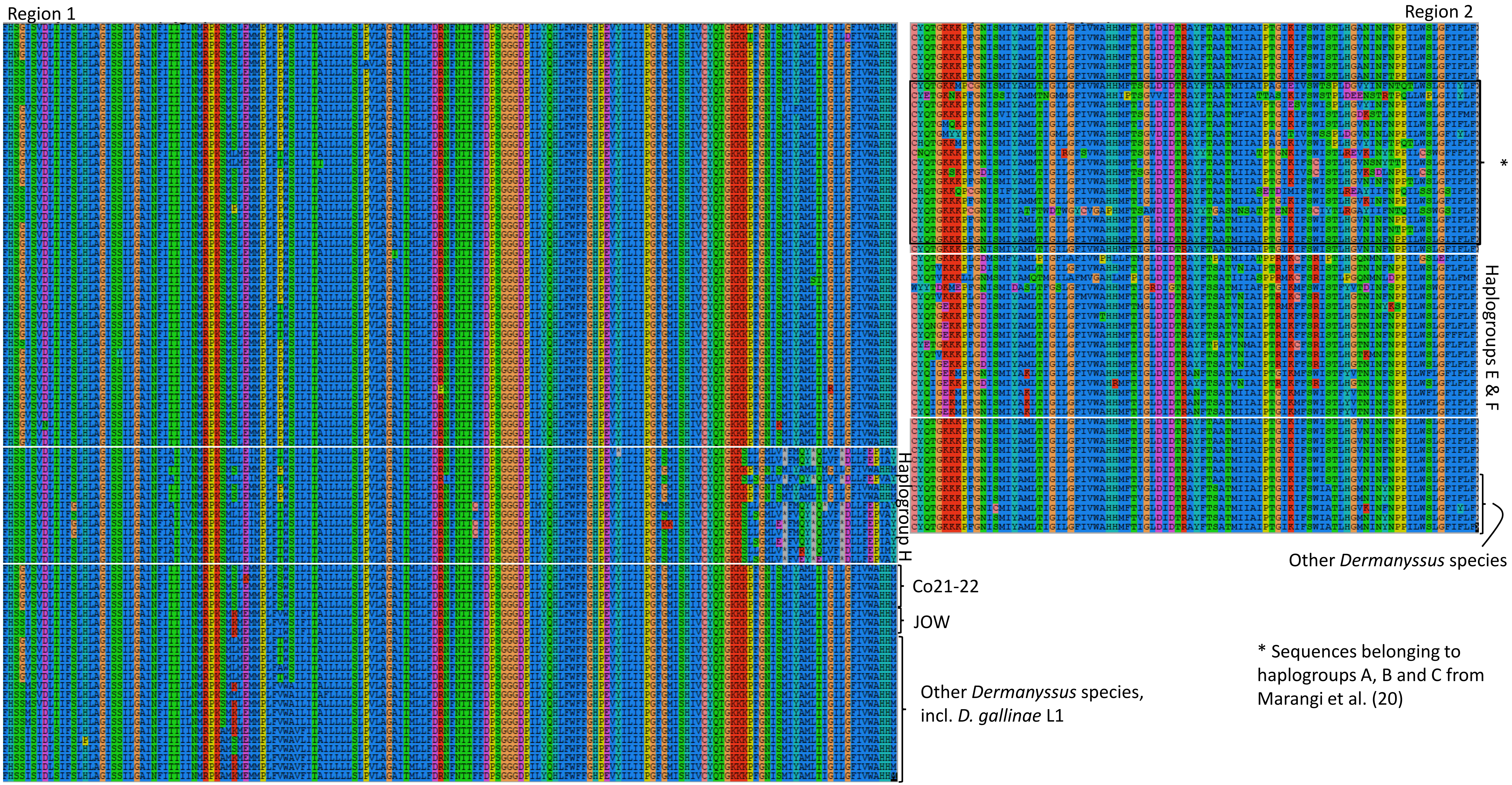

Supplementary material 3. **Heatmap of the frequencies of Co1 haplogroups and Tpm alleles within the isolates** (= large samples from single farm buildings or groups of nests) **studied by Roy & Buronfosse (4), Cilogclu et al. (24) and in the present study, as well as diversity indices.** *Other T,* Tpm alleles detected other than the 4 previous ones; N, number of sequenced individuals; S, number of segregating sites; h, number of haplotypes; Hd, haplotype diversity, K, average number of differences; PiJC, nucleotide diversity with JC correction. Mitochondrial (mt) profiles are delineated as follows: single typical, a single typical haplogroup in the farm building; two typical, two typical haplogroups; atypical, one or more atypical haplogroups; atypical + typical, one or more atypical haplogroups + a typical haplogroup (low frequency).

Study	Species	Isolate	Country (area)	Habitat	Sampling year	*Frequency of mt Co1 haplogroups*							*Frequency of nuclear Tpm alleles*						N	S	h	Hd	K	PiJC	mt profile in D. gallinae s.s. populations
						L1	A	B	C	E	F	H	L1	Tro_1	Tro_2	Tro_3	Tro_17	Other T							
Roy & Buronfosse (2011)	*D. gallinae* L1	9001	France (E)	Pigeon farm	2009	1.00	0.00	0.00	0.00	0.00	0.00	0.00	1.00	0.00	0.00	0.00	0.00	0.00	-	-	-	-	-	-	-
	D. gallinae L1	L1FRfarm	France	Pigeon farm	2007-2008	1.00	0.00	0.00	0.00	0.00	0.00	0.00	1.00	0.00	0.00	0.00	0.00	0.00	-	-	-	-	-	-	-
	D. gallinae L1	L1FRwild	France	Wild pigeons	2009	1.00	0.00	0.00	0.00	0.00	0.00	0.00	1.00	0.00	0.00	0.00	0.00	0.00	-	-	-	-	-	-	-
	D. gallinae L1	L1US	USA	Pigeon farm	2009	1.00	0.00	0.00	0.00	0.00	0.00	0.00	1.00	0.00	0.00	0.00	0.00	0.00	-	-	-	-	-	-	-
	D. gallinae s.s.	IL	The Netherlands	Wild starlings	2007	0.00	1.00	0.00	0.00	0.00	0.00	0.00	0.00	0.06	0.00	0.00	0.00	0.94	16	3	4	0.725	0.950	0.002	single typical
	D. gallinae s.s.	ROL	France (SE)	Wild rollers	2008	0.00	1.00	0.00	0.00	0.00	0.00	0.00	0.00	0.00	0.00	0.08	0.00	0.92	18	3	2	0.209	0.627	0.001	single typical
	D. gallinae s.s.	8019	France (SE)	Layer farm	2008	0.00	0.05	0.00	0.95	0.00	0.00	0.00	0.00	0.05	0.95	0.00	0.00	0.00	20	25	4	0.432	2.668	0.005	several typical
	D. gallinae s.s.	8020	France (NW)	Layer farm	2008	0.00	0.00	0.12	0.88	0.00	0.00	0.00	0.00	0.39	0.44	0.14	0.00	0.03	17	21	3	0.412	4.632	0.009	several typical
	D. gallinae s.s.	8021	France (E)	Layer farm	2008	0.00	0.91	0.05	0.05	0.00	0.00	0.00	0.00	0.09	0.50	0.18	0.09	0.14	22	32	3	0.177	3.736	0.007	several typical
	D. gallinae s.s.	8022	France (NW)	Layer farm	2008	0.00	0.00	0.00	1.00	0.00	0.00	0.00	0.00	0.16	0.34	0.50	0.00	0.00	19	0	1	0.000	0.000	0.000	single typical
	D. gallinae s.s.	8028	France (E)	Layer farm	2008	0.00	0.00	0.60	0.40	0.00	0.00	0.00	0.00	0.05	0.65	0.28	0.00	0.03	20	24	3	0.542	11.721	0.023	several typical
	D. gallinae s.s.	8029	France (E)	Layer farm	2008	0.00	0.00	0.52	0.48	0.00	0.00	0.00	0.00	0.12	0.33	0.50	0.00	0.05	21	25	3	0.657	12.581	0.024	several typical
	D. gallinae s.s.	9003	France (E)	Layer farm	2008	0.00	1.00	0.00	0.00	0.00	0.00	0.00	0.00	0.10	0.60	0.31	0.00	0.00	21	6	2	0.495	2.971	0.006	single typical
	D. gallinae s.s.	9004	France (E)	Layer farm	2009	0.00	1.00	0.00	0.00	0.00	0.00	0.00	0.00	0.50	0.46	0.04	0.00	0.00	23	7	2	0.443	3.099	0.006	single typical
	D. gallinae s.s.	9005	France (E)	Layer farm	2009	0.00	0.00	0.00	1.00	0.00	0.00	0.00	0.00	0.05	0.52	0.36	0.00	0.07	22	0	1	0.000	0.000	0.000	single typical
	D. gallinae s.s.	9007	France (E)	Layer farm	2009	0.00	0.38	0.00	0.62	0.00	0.00	0.00	0.00	0.08	0.53	0.23	0.14	0.03	37	27	4	0.521	12.170	0.024	several typical
	D. gallinae s.s.	9016	France (E)	Layer farm	2009	0.00	0.94	0.00	0.06	0.00	0.00	0.00	0.00	0.11	0.47	0.21	0.03	0.18	33	26	3	0.174	2.996	0.006	several typical
	D. gallinae s.s.	AUS	Australia	Layer farm	2009	0.00	1.00	0.00	0.00	0.00	0.00	0.00	0.00	0.96	0.00	0.04	0.00	0.00	24	3	3	0.236	0.402	0.001	single typical
	D. gallinae s.s.	BOUY	France (SE)	Layer farm	2009	0.00	1.00	0.00	0.00	0.00	0.00	0.00	0.00	0.38	0.44	0.18	0.00	0.00	17	6	4	0.566	1.368	0.003	single typical
	D. gallinae s.s.	BREa	Brazil	Layer farm	2009	0.00	1.00	0.00	0.00	0.00	0.00	0.00	0.00	0.13	0.00	0.73	0.00	0.15	20	0	1	0.000	0.000	0.000	single typical
	D. gallinae s.s.	BREb	Brazil	Layer farm	2009	0.00	1.00	0.00	0.00	0.00	0.00	0.00	0.00	0.00	0.00	0.92	0.00	0.08	18	1	2	0.111	0.111	0.000	single typical
	D. gallinae s.s.	PO	Poland	Layer farm	2007	0.00	0.00	1.00	0.00	0.00	0.00	0.00	0.00	0.15	0.32	0.53	0.00	0.00	17	2	2	0.515	1.029	0.002	single typical
	D. gallinae s.s.	REN	France (NW)	Layer farm	2009	0.00	0.00	0.00	1.00	0.00	0.00	0.00	0.00	0.00	0.93	0.08	0.00	0.00	20	3	4	0.363	0.389	0.001	single typical
	D. gallinae s.s.	SK	(origin Denmark)	Lab system	2009	0.00	1.00	0.00	0.00	0.00	0.00	0.00	0.00	0.26	0.64	0.10	0.00	0.00	21	3	4	0.348	0.371	0.001	single typical
Ciloglu et al. (2020)	*D. gallinae* s.s.	Kayseri1	Turkey	Layer farm	2016-2017	0.00	0.04	0.00	0.00	0.07	0.89	0.04	-	-	-	-	-	-	27	60	6	0.806	8.598	0.034	atypical + typical
	D. gallinae s.s.	Kayseri2	Turkey	Layer farm	2016-2017	0.00	0.00	0.00	0.00	0.10	0.90	0.00	-	-	-	-	-	-	10	32	4	0.778	7.356	0.029	atypical
	D. gallinae s.s.	Kayseri3	Turkey	Layer farm	2016-2017	0.00	0.04	0.00	0.00	0.12	0.85	0.04	-	-	-	-	-	-	26	60	6	0.825	10.652	0.042	atypical + typical
	D. gallinae s.s.	Nevsehir4	Turkey	Layer farm	2016-2017	0.00	0.00	0.00	0.00	0.00	1.00	0.00	-	-	-	-	-	-	17	4	4	0.757	1.721	0.006	atypical
	D. gallinae s.s.	Nevsehir5	Turkey	Layer farm	2016-2017	0.00	0.00	0.00	0.00	0.00	1.00	0.00	-	-	-	-	-	-	20	4	4	0.784	1.674	0.006	atypical
Present	*D. gallinae* s.s.	BE01	Belgium	Layer farm	2020	0.00	0.00	1.00	0.00	0.00	0.00	0.00	-	-	-	-	-	-	18	15	5	0.712	4.922	0.009	single typical
	D. gallinae s.s.	BE02	Belgium	Layer farm	2020	0.00	0.00	0.18	0.82	0.00	0.00	0.00	-	-	-	-	-	-	16	24	3	0.342	5.492	0.011	several typical
	D. gallinae s.s.	FR01	France (NW)	Layer farm	2020	0.00	0.00	0.00	1.00	0.00	0.00	0.00	-	-	-	-	-	-	20	0	1	0.000	0.000	0.000	single typical
	D. gallinae s.s.	FR02	France (NW)	Layer farm	2020	0.00	0.00	0.00	1.00	0.00	0.00	0.00	-	-	-	-	-	-	20	3	3	0.195	0.300	0.001	single typical
	D. gallinae s.s.	FRNP01	France (SE)	Layer farm	2020	0.00	0.00	0.00	1.00	0.00	0.00	0.00	-	-	-	-	-	-	14	0	1	0.000	0.000	0.000	single typical
	D. gallinae s.s.	DIN	France (SE)	Layer farm	2020	0.00	0.00	0.00	1.00	0.00	0.00	0.00	0.00	0.33	0.52	0.14	0.00	0.00	24	1	2	0.083	0.083	0.000	single typical
	D. gallinae s.s.	GZ	France (SE)	Backyard henh	2018	0.00	0.00	0.00	1.00	0.00	0.00	0.00	-	-	-	-	-	-	24	0	1	0.000	0.000	0.000	single typical
	D. gallinae s.s.	JOUV	France (SE)	Layer farm	2019	0.00	0.00	0.00	1.00	0.00	0.00	0.00	0.00	0.00	0.50	0.50	0.00	0.00	24	0	1	0.000	0.000	0.000	single typical
	D. gallinae s.s.	SPT_2018	France (SE)	Layer farm	2018	0.00	0.00	0.00	1.00	0.00	0.00	0.00	-	-	-	-	-	-	10	3	4	0.533	0.600	0.001	single typical
	D. gallinae s.s.	SPT_2020	France (SE)	Layer farm	2020	0.00	0.00	0.00	1.00	0.00	0.00	0.00	-	-	-	-	-	-	16	1	2	0.125	0.125	0.000	single typical
	D. gallinae s.s.	CRZA	France (N)	Layer farm	2018	0.00	0.00	0.00	1.00	0.00	0.00	0.00	-	-	-	-	-	-	14	0	1	0.000	0.000	0.000	single typical
	D. gallinae s.s.	H	France (SW)	Layer farm	2018	0.00	0.00	0.00	0.06	0.00	0.00	0.94	0.00	0.00	0.54	0.46	0.00	0.00	16	77	14	0.983	23.825	0.047	atypical + typical

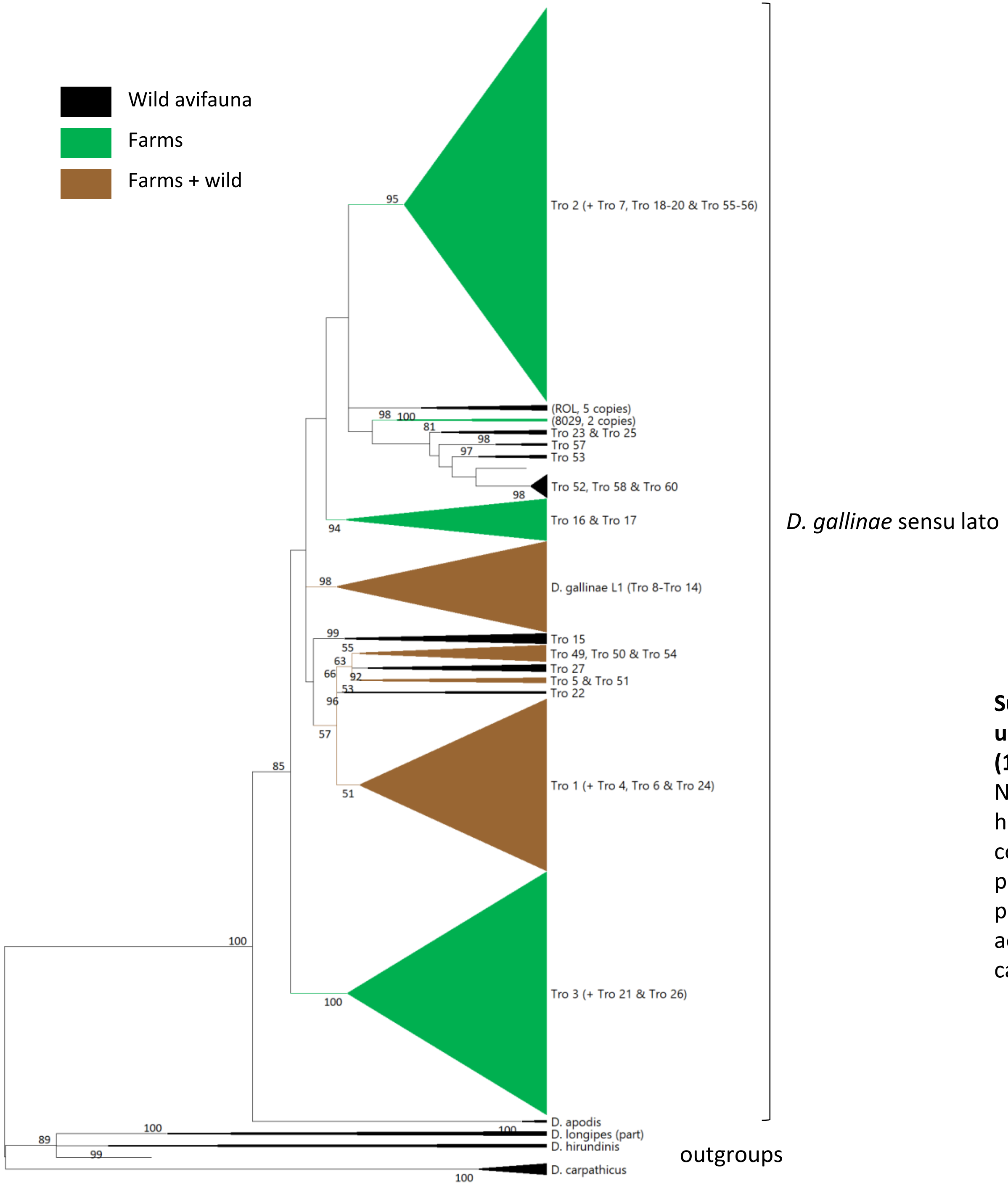

Supplementary material 4. Evolutionary history of Tpm alleles (nuclear DNA) recorded from *Dermanyssus* until now, ie from Roy et al. (2), Roy & Buronfosse (4) and the homozygous sequences from present data (1026 nucleotide sequences; total of 735 positions in the final dataset). It was inferred by using the Tamura-Nei model with a discrete Gamma distribution (5 categories (+*G*, parameter = 0.5196)). The tree with the highest log likelihood (-2961.59) is shown. Only bootstrap values >50% are displayed at the nodes. The clades corresponding to the main allele groups are condensed into triangles according to the following rules: width proportional to the number of occurrences in the populations analysed in Roy & Buronfosse (4) and in the present study, grouping by alleles identified according to the denominations 'Tro n' of Roy et al. (2), color according to the origin of the individuals carrying the allele group. On farms, most heterozygous individuals carry two distant alleles, belonging respectively to the clades Tro 1, Tro 2 or Tro 3.
